# Genetic Fingerprint of *Klebsiella pneumoniae* Virulence: A Systematic Review

**DOI:** 10.3390/pathogens15050556

**Published:** 2026-05-21

**Authors:** Carlos Andrés Aldana-Ortega, Alexander José Pérez-Villadiego, Yohelys Monterrosa-Taborda, Alberto Angulo-Ortíz, Orfa Inés Contreras-Martínez

**Affiliations:** 1 Biology Department, Faculty of Basic Sciences, University of Córdoba, Montería 230002, Colombia; caldanaortega71@correo.unicordoba.edu.co (C.A.A.-O.); aperezvilladiego49@correo.unicordoba.edu.co (A.J.P.-V.); ymonterrosataborda64@correo.unicordoba.edu.co (Y.M.-T.); 2 Chemistry Department, Faculty of Basic Sciences, University of Córdoba, Montería 230002, Colombia; aaangulo@correo.unicordoba.edu.co

**Keywords:** *Klebsiella pneumoniae*, hypervirulent, genes, virulence determinants, molecular techniques

## Abstract

Background: *Klebsiella pneumoniae* is a globally relevant pathogen whose growing association between hypervirulence and antimicrobial resistance represents a major public health challenge. Methods: A systematic review was performed following the PRISMA 2020 guidelines. Studies published between 2005 and 2025 were searched in Google Scholar, Scopus, PubMed and Science Direct that reported the molecular detection of virulence genes in clinical isolates. Results: A total of 676 studies were included, in which 475 virulence genes were reported. A progressive increase in their detection was observed, Hypervirulent strains were associated with a higher proportion of genes associated with capsule and hypermucoviscosity, while classical strains were associated with a higher representation of adhesion and biofilm genes. Conclusions: The virulence of *K. pneumoniae* is organized into functional modules dominated by iron acquisition and capsular regulation. These findings support the prioritization of key determinants for molecular surveillance and the study of the global distribution and temporal trends of this pathogen.

## 1. Introduction

*Klebsiella pneumoniae* is a Gram-negative bacillus of notable clinical relevance. This microorganism is responsible for a broad spectrum of both nosocomial and community-acquired infections, including pneumonia, urinary tract infections, liver abscesses, and severe sepsis. Its impact is especially critical in vulnerable populations such as neonates, older adults, and immunocompromised patients [1,2]. This microorganism is recognized as one of the main agents of the ESKAPEE group (*Enterococcus faecium*, *Staphylococcus aureus*, *Klebsiella pneumoniae*, *Acinetobacter baumannii*, *Pseudomonas aeruginosa*, *Enterobacter* spp., and *Escherichia coli*), which represent a serious threat to global public health due to their high antimicrobial resistance and high pathogenicity [3]. The World Health Organization (WHO) has classified carbapenemase-producing pathogens, including *K. pneumoniae*, as critical priority pathogens [4].

The ability of *K. pneumoniae* to cause a wide range of infections is largely attributed to the genetic diversity of its virulence factors, which include a capsule that confers resistance to phagocytosis and a lipopolysaccharide (LPS) with endotoxic function that reinforces this protection; type I and III fimbriae, which allow adherence to host cells and facilitate infection; and siderophores or iron acquisition systems, among others; whose variability directly influences their clinical and epidemiological behavior [5]. Many of these virulence factors are located in mobile genetic elements and can be transferred horizontally via plasmids, integrative genomic islands, and other elements, increasing the virulence potential of bacteria and favoring the emergence of strains with convergent resistance and virulence profiles [6]. Taken together, these evolutionary changes in virulence determinant genes have contributed to a wide genomic diversity, reflecting the adaptation of *K. pneumoniae* to different hospital niches [7].

Since the 1980s, with the introduction of the polymerase chain reaction (PCR), advances in molecular and genomic techniques have profoundly transformed our understanding of bacterial virulence. The identification and characterization of virulence factors in pathogens are crucial for understanding the pathogenesis of the diseases they cause, allowing for the detection of specific virulence genes [8]. Although there are reviews on the biology of virulence, epidemiology and emergence of *K. pneumoniae*, a systematic and quantitative synthesis of how the main genetic determinants of virulence in clinical isolates have evolved globally over the last two decades, in terms of prevalence and geographical distribution, has not yet been carried out. Despite the growing body of evidence, the available knowledge remains fragmented, largely because most studies have focused on very specific contexts, such as intensive care units, isolated hospital outbreaks, or certain geographical regions [9,10,11,12]. This gap limits the possibility of comparing and exploring the relationship between sample type, patient, strain and their virulence profiles.

Additionally, it is important to consider that the increase in the identification of virulence genes reported in the literature over recent decades has been closely influenced by the development and adoption of more sensitive, high-resolution molecular techniques, rather than by a direct biological evolution of these determinants. In this regard, the patterns observed in the literature primarily reflect trends in the detection and reporting of virulence genes, shaped by the methodological approaches employed.

In this context, the present systematic review aims to analyze trends in the identification and reporting of *K. pneumoniae* virulence factors detected using molecular techniques in clinical isolates reported globally over the past 20 years. Specifically, we aim to: (i) identify and classify the main virulence genes and determinants of *K. pneumoniae* described in the literature between 2005 and 2025, according to the molecular methodologies used; (ii) assess the frequency and geographic distribution of these genes, differentiating by region, type of clinical sample, and hospital setting; and (iii) describe temporal trends and patterns in the detection and reported diversity of virulence factors over the last two decades.

By systematically integrating and comparing the available evidence, this review seeks to provide a comprehensive and up-to-date overview of virulence gene reporting patterns, serving as a foundation for strengthening genomic surveillance strategies, informing the design of clinical studies, and supporting policy development for the control of this priority pathogen.

## 2. Materials and Methods

### 2.1. Systematic Review

We conducted a systematic review focused on the identification and reporting patterns of virulence-associated genes in *K. pneumoniae*, following the guidelines outlined in the Preferred Reporting Items for Systematic Reviews and Meta-Analyses (PRISMA 2020) [13,14]. The PRISMA 2020 checklist is provided as Supplementary Material (Table S1).

### 2.2. Search Strategy

A comprehensive literature search was conducted to identify studies reporting genes associated with virulence determinants in *K. pneumoniae*, detected using molecular techniques in both clinical isolates and community-acquired infections. The search was performed in the following databases: Google Scholar, Scopus, PubMed, and ScienceDirect, up to 28 September 2025. Google Scholar was included to increase search sensitivity and capture potentially relevant studies not indexed in other databases. To minimize bias, all records were systematically screened, duplicates were removed using Zotero, and only peer-reviewed studies meeting strict eligibility criteria were included.

Search strategies were developed using combinations of Medical Subject Headings (MeSH) and free-text terms, adapted to each database, and combined using Boolean operators (“AND”, “OR”). The main search string included: (“*Klebsiella pneumoniae*” OR “*K. pneumoniae*”) AND (“virulence” OR “virulence genes” OR “virulence factors” OR “pathogenicity” OR “virulence determinants” OR “hypermucoviscosity”) AND (“molecular techniques” OR “molecular methods” OR “molecular epidemiology” OR “genome sequencing” OR “genotyping” OR “molecular characterization”).

Additionally, complementary terms related to major virulence factors were incorporated, including “capsule”, “capsular polysaccharide”, “fimbriae”, “type 1 fimbriae”, “type 3 fimbriae”, “adhesins”, “siderophores”, “enterobactin”, “aerobactin”, “yersiniabactin”, “salmochelin”, “biofilm formation”, and “lipopolysaccharide”. These terms were applied to titles, abstracts, and keywords to maximize search sensitivity. The complete search strategies for each database, including full search strings, applied filters, and the number of records retrieved, are provided in Supplementary Materials, Table S2. Database search.

**Figure 1:**
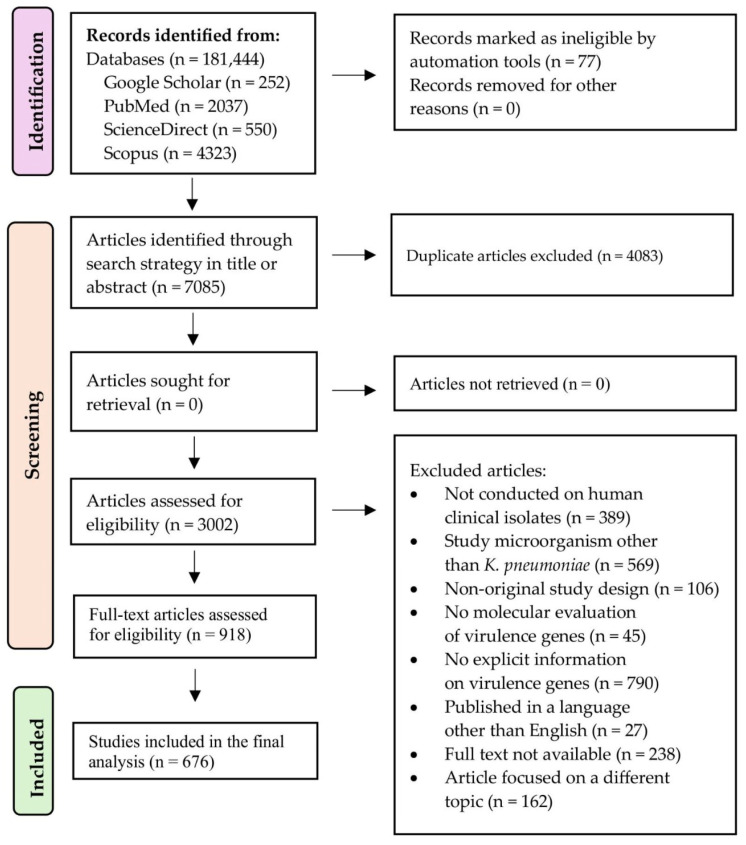
PRISMA flow diagram of study identification, screening, and inclusion.

Time filters, language filters for English, and a filter for grey literature were applied when the databases allowed it. The years, titles, abstracts, countries, number of clinical isolates, strain types, sources of isolates, patient types, and indexing terms of the articles were also examined. All records were managed using Zotero 7.0.32 (64-bit), then exported to Microsoft Excel (2025). Microsoft 365 (version 2512) [Software], where duplicates were removed. The stepwise selection of studies was performed following the PRISMA 2020 recommendations, Figure 1.

### 2.3. Eligibility Criteria

We included only studies published in English. The study population comprised individuals with hospital-acquired or community-acquired infections. We required that studies document the detection of virulence genes using molecular techniques (e.g., whole-genome sequencing, high-throughput sequencing, multiplex PCR, real-time PCR, uniplex PCR, RT-PCR). Regarding outcomes, we considered the frequency of detection and reporting patterns of virulence genes documented globally over the past 20 years as part of the eligibility criteria; specifically, studies had to report at least one virulence gene frequency. This is a descriptive observational systematic review. Eligible study designs included case reports, case series, cohort analyses, case–control studies, and exposure trials (both randomized and non-randomized) that presented outcome data.

### 2.4. Study Selection

The selection of studies was carried out independently by two reviewers (C.A.A.O., A.J.P.V.); discrepancies between them were resolved by a third reviewer (Y.M.T.). Initially, the selection of all articles was carried out by reviewing titles and abstracts. Any article that clearly did not meet the inclusion criteria or that was outside the scope of *K. pneumoniae* infections was excluded. To ensure rigorous selection, full texts were analyzed. These full texts of all relevant articles were then reviewed in detail by the same review team. Exclusion criteria were also recorded, which included: non-original articles, articles in languages other than English, studies conducted on non-clinical (environmental or animal) strains, species other than *K. pneumoniae*, studies without evaluation by molecular techniques, and those for which the full text was unavailable.

### 2.5. Data Extraction

Data extraction was performed independently by two reviewers (C.A.A.O., A.J.P.V.) using a standardized matrix. Discrepancies were resolved by consensus with a third reviewer (Y.M.T.). A standardized matrix was used in Microsoft Excel (2025). Microsoft 365 (version 2512) was used for data extraction to collect information on: study design, year of study, country, number of isolates per study, strain type, clinical origin, patient type, molecular technique used, and virulence-associated genes detected. Any discrepancies in data extraction were resolved by group consensus.

#### 2.5.1. Study Variables and Outcomes

The predefined outcomes of this review included the presence of virulence genes in clinical isolates of *K. pneumoniae*, the frequency of detection of each gene across included studies, and reporting patterns according to time period, strain type (classic versus hypervirulent), clinical origin, and geographic distribution. Additional variables extracted from each study included study design, year of publication, country, number of isolates, strain type, clinical source, patient population (adult, pediatric, or neonatal), and molecular detection method.

Each included study constituted the primary unit of analysis. When multiple genes or isolates were reported within a single study, each gene detection was considered independently for descriptive frequency analyses. Frequencies were calculated as the proportion of studies reporting a given virulence gene relative to the total number of studies assessing that gene or functional category. To ensure conceptual accuracy, these measures are referred to as reporting frequencies rather than epidemiological prevalence, as they reflect detection patterns within the published literature rather than true population-level prevalence.

Regarding missing data, no imputation or assumptions were applied. Studies with incomplete reporting of specific variables were included in all analyses for which relevant data were available and excluded only from subgroup analyses requiring the missing information. For instance, studies reporting gene detection without specifying strain type contributed to overall gene frequency estimates but were not included in analyses stratified by strain classification.

#### 2.5.2. Effect Measures

For each virulence category (e.g., siderophores, adhesion, and capsule), the detection frequency of each individual gene was calculated as the proportion of studies reporting that gene relative to the total number of studies that assessed the corresponding functional category. This approach allowed comparison of the most frequently reported genes within each functional group. For each virulence category (e.g., siderophores, adhesion, and capsule), the detection frequency of each individual gene was calculated as the proportion of studies reporting that gene relative to the total number of studies that assessed the corresponding functional category. This approach allowed comparison of the most frequently reported genes within each functional group. Owing to the heterogeneity among the included studies and the descriptive nature of this level of analysis, results are presented as reporting frequencies, and no formal meta-analysis was performed.

To further explore factors influencing gene reporting, the five most frequently reported virulence genes across all included studies (*rmpA*, *rmpA2*, *mrkD*, *iucA*, and *iutA*) were selected for comparative subgroup analyses. These genes were chosen because they represented the most consistently reported determinants among capsule-associated, adhesion, and siderophore-related virulence mechanisms.

First, comparative analyses were performed according to strain type (cKp vs. hvKp). For these analyses, the denominator used for each proportion corresponded to the total number of studies that explicitly reported the respective strain classification. Studies lacking strain typing information were excluded from these specific comparisons. When the same study reported the presence of the same gene in both subgroups, observations were counted independently within each category.

Second, molecular techniques reported across studies were grouped into two analytical categories: PCR-based methods (conventional PCR, multiplex PCR, RT-qPCR, qPCR, RT-PCR, and LAMP) and sequencing-based methods (WGS, NGS, mNGS, RNA-seq, and MLST). Comparative analyses were then performed to evaluate whether the frequency of gene detection differed according to the molecular method used. For these analyses, the denominator corresponded to the total number of studies included in each methodological group.

For both comparative analyses, differences in proportions between groups were estimated together with their corresponding 95% confidence intervals (95% CI) using the Newcombe–Wilson method with continuity correction. Statistical significance between groups was assessed using Fisher’s exact test, considering a two-sided *p* value < 0.05 as statistically significant. All analyses were performed using GraphPad Prism version 8.0.2 (263) (GraphPad Software, San Diego, CA, USA). Details of the data used for the analyses are provided as Supplementary Material, Table S3.

#### 2.5.3. Risk of Bias Assessment

Two reviewers (C.A.A.O., A.J.P.V.) independently assessed the methodological quality and risk of bias of each included study using EMMO-Vir (Molecular Methodological Assessment for Virulence Studies). This tool was developed by the authors specifically for the present review, as no validated or standardized instrument adequately addresses the methodological domains relevant to molecular epidemiology studies of bacterial virulence genes (e.g., primer design validity, molecular technique reporting, and virulence gene definitions). EMMO-Vir was developed based on established methodological frameworks, including STROBE [15], STARD [16], QUADAS-2 [17], and JBI criteria [18], and was refined through an internal iterative review process to improve item clarity and applicability before full assessment. The tool comprises 22 items distributed across four domains (Supplementary Table S4): (1) Methodological Quality (3 items), (2) Technical Quality (9 items), (3) Methodology and Bias (5 items), and (4) Interpretation and Transparency (5 items). Each item was scored as “Yes” (1 point), “No” (0 points), or “Not applicable”. An overall percentage score was calculated as (total points/total applicable items) × 100, and studies were classified as high quality (86–100%), moderate quality (66–85%), or low quality (0–65%). Discrepancies between reviewers were resolved by consensus with a third reviewer (Y.M.T.). Risk of bias was assessed at the study level, and detailed domain-specific scores are provided in Supplementary Table S5.

Although EMMO-Vir has not undergone external validation, its development was informed by established methodological frameworks and is reported transparently to support reproducibility and future validation in similar molecular epidemiological studies.

#### 2.5.4. Grouping Criteria for the Analyses

The studies were grouped for analysis according to the following criteria: time period (2005–2010, 2011–2015, 2016–2020, 2021–2025), strain type (classical cKp or hypervirulent hvKp), clinical origin (blood, urinary tract, respiratory tract, etc.), and geographic region (Asia, Europe, America, Africa, Oceania). All included studies contributed to all syntheses for which the necessary data were available. Additional clinically relevant variables, such as infection status (symptomatic versus colonization), patient condition (e.g., immunocompromised status or surgical context), and healthcare setting (ICU versus non-ICU), were not included as grouping criteria due to their inconsistent and limited reporting across studies, which precluded meaningful comparative analyses. Additionally, subgroup analyses were performed for a subset of the most frequently reported virulence-associated genes (*rmpA*, *rmpA2*, *mrkD*, *iucA*, and *iutA*), as described in Section 2.5.2, to explore differences according to strain type and molecular detection methods.

#### 2.5.5. Handling Missing Data

Missing data were not imputed. Studies with incomplete information for a given variable were excluded only from the analysis of that specific variable. Given the heterogeneity in study designs and reporting practices, this approach ensured consistency across analyses while avoiding artificial data assumptions.

#### 2.5.6. Presentation of Results

The results are presented using tables of absolute and relative frequencies (percentages), comparative statistical tables, graphs, and a choropleth map.

### 2.6. Statistical Analysis

For the synthesis of the results, descriptive statistics were performed using absolute frequencies, generating comparative graphs and tables.

Descriptive statistics were used to summarize the absolute and relative frequencies of virulence genes across the included studies, generating comparative tables, graphical representations, and subgroup distributions. For selected subgroup comparisons, differences in proportions were estimated together with their corresponding 95% confidence intervals (95% CI), and statistical significance between groups was evaluated using Fisher’s exact test. All statistical analyses were conducted using GraphPad Prism version 8.0.2 (263) (GraphPad Software, San Diego, CA, USA).

#### 2.6.1. Narrative Summary of Results

A meta-analysis was not performed due to substantial clinical and methodological heterogeneity across studies, including differences in study design, sample size, molecular techniques (ranging from PCR to WGS), and inconsistent definitions of cKp and hvKp over time. Additional clinically relevant variables—such as infection status (symptomatic vs. colonization), patient characteristics, and healthcare setting—were also reported inconsistently, precluding meaningful subgroup comparisons. Therefore, a narrative and descriptive synthesis was conducted, reporting the frequencies and percentages of the virulence genes and functional groups identified.

#### 2.6.2. Heterogeneity Analysis

Heterogeneity was explored through descriptive subgroup analyses, with results stratified by time period, strain type (cKp vs. hvKp), clinical origin, and geographic region. For selected highly reported virulence genes, subgroup comparisons between strain types were further quantified by estimating differences in reporting proportions and their 95% confidence intervals. This approach is consistent with the exploratory nature of the review and the characteristics of the available data.

#### 2.6.3. Sensitivity Analysis

Sensitivity analyses were not performed due to the absence of a meta-analysis.

#### 2.6.4. Bias Assessment

Potential publication bias was assessed qualitatively by comparing reported outcomes with study objectives. Due to substantial heterogeneity in study designs, gene targets, and reporting practices, a formal assessment of selective reporting was not possible.

Therefore, the analysis was limited to explicitly reported virulence genes, and the absence of specific genes was not interpreted as true absence, but as unreported data, which may influence the observed frequency and diversity of virulence determinants.

### 2.7. PROSPERO Registration

This systematic review was not prospectively registered. However, it was registered in the International Prospective Register of Systematic Reviews (PROSPERO) following completion of the literature search and data extraction. The registration number is CRD420251251276, and the record was published on 11 December 2025. The protocol is available at: https://www.crd.york.ac.uk/PROSPERO/view/CRD420251251276 (accessed on 11 December 2025). The protocol and any material related to this review are available. No subsequent amendments were made to the registered protocol.

### 2.8. Ethical Approval

The analysis in this review was limited to data from previously published research; therefore, ethical approval was not required.

## 3. Results

### 3.1. Systematic Review and Characteristics of Included Studies

**Figure 2:**
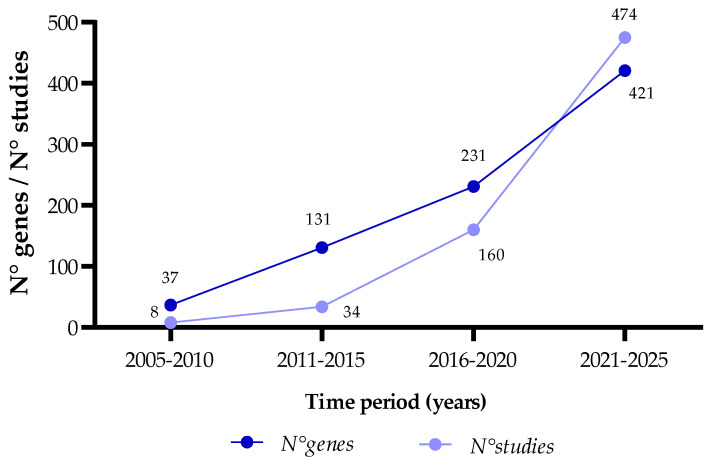
Temporal trends in the number of studies and virulence-associated genes reported for *K. pneumoniae* across the evaluated time periods (2005–2025). The blue line represents the number of genes identified, while the light blue line indicates the number of studies.

**Figure 3:**
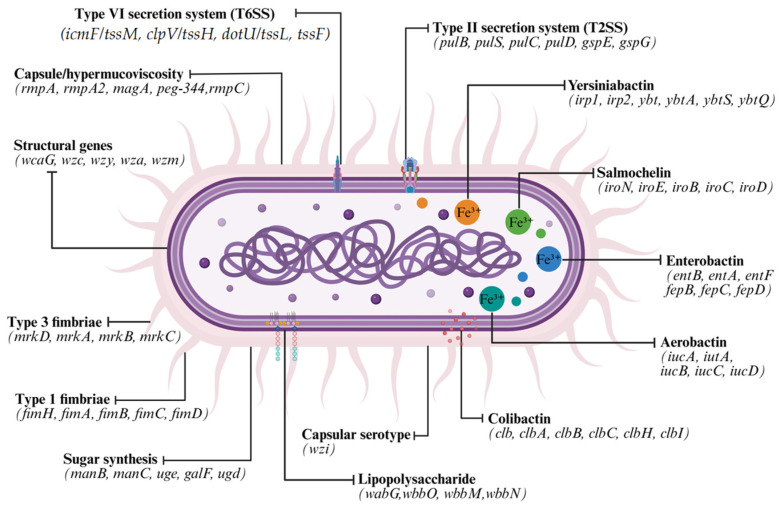
Virulence-associated genes of *K. pneumoniae*. Schematic overview of the main genetic determinants associated with virulence in *K. pneumoniae*. These include genes involved in capsule formation and hypermucoviscosity (capsular locus structural components, regulatory genes, and serotype-associated genes); iron acquisition systems and siderophores (enterobactin, aerobactin, yersiniabactin, and salmochelin); adhesion and biofilm formation mediated by type 1 and type 3 fimbriae; lipopolysaccharide-associated components; pathways related to sugar biosynthesis; type II (T2SS) and type VI (T6SS) secretion systems; and the colibactin gene cluster.

Detailed study-level results, including the detection and positivity percentages of virulence-associated genes for each included study, are presented in Supplementary Materials, Table S6. A total of 7162 records were identified by searching the four databases, applying a title and abstract filter. After removing 4083 duplicates and excluding 77 records that could not be retrieved, 3002 records were reviewed by title and abstract. Of these, 2084 records were excluded for not meeting the inclusion criteria. The eligibility of a total of 918 full-text articles was assessed. Of these, 242 articles were excluded for predefined reasons (see Supplementary Materials, Table S7). Finally, 676 studies were included in the systematic review. The study selection process is summarized in Figure 1 (PRISMA 2020 flowchart). The reasons for exclusion were: that were not clinical isolates in humans (n = 389), that the microorganism was different from *K. pneumoniae* (n = 569), previously published reviews and meta analyses (n = 106), articles that did not evaluate the molecular detection of virulence genes (n = 45), articles that did not have the full text (n = 238), articles in languages other than English (n = 27), articles with different themes (n = 162) and studies that lacked data on virulence genes (n = 790). Finally, this review included 676 studies published between 2005 and 2025, which collectively identified a total of 475 genes associated with the virulence and pathogenicity of *K. pneumoniae*, identified using various molecular techniques. Throughout the four periods evaluated, an increase was observed in the volume of publications, the diversity of reported genes, and the use and evolution of molecular techniques employed (Figure 2). A summary of the temporal evolution of these genes is shown in Table 1. In addition, the major virulence associated genetic determinants identified across the included studies are schematically summarized in Figure 3.

### 3.2. Genes Reported in the Period 2005–2010

A total of 8 studies reported 37 virulence-associated genes in *K. pneumoniae* (Table 2). Despite the limited number of studies, a structured distribution between classical (cKp, 54%) and hypervirulent strains (hvKp, 46%) was already evident. All detections were performed using conventional PCR, indicating that the observed repertoire reflects targeted rather than comprehensive detection.

Clinical origin showed a heterogeneous distribution. While isolates were mainly associated with the digestive system (44%), stratification revealed a clear divergence: cKp was exclusively linked to urinary tract infections, whereas hvKp exhibited a broader distribution across digestive, bloodstream, urinary, and respiratory infections. A similar pattern was observed in demographics, with hvKp detected only in adults, while cKp was distributed between adults and neonates.

At the functional level, virulence genes were unevenly distributed and concentrated in specific groups. Adhesion factors predominated, particularly type 1 (21.43%) and type 3 fimbriae (11.90%), followed by capsule-associated determinants (19.05%), including regulators of hypermucoviscosity. Structural and biosynthetic capsule genes were also consistently detected. In contrast, iron acquisition systems and LPS-related genes showed low frequencies, whereas T6SS displayed a relatively higher contribution (16.67%).

Stratification by strain type revealed distinct profiles. In cKp, virulence was dominated by adhesion (45%) and T6SS (35%), with minor contributions from other factors. In hvKp, the profile was more concentrated in capsule-related functions and outer envelope components, along with a relevant contribution of type 3 fimbriae.

Overall, this period shows an early functional differentiation, with cKp associated mainly with adhesion-related mechanisms and hvKp with capsule-associated determinants and broader clinical distribution.

### 3.3. Genes Reported in the Period 2011–2015

Thirty-four studies were identified, documenting 131 genes associated with the virulence and pathogenicity of *K. pneumoniae* (Table 3). Compared to the previous period, this represents a marked expansion in the number and diversity of reported genes, indicating a broader exploration of the virulence repertoire. Although conventional PCR remained the predominant technique (67.65%), the incorporation of whole-genome sequencing (17.65%) and other molecular approaches suggests the beginning of a methodological transition that likely contributed to the detection of a more diverse set of genes.

The distribution of clinical origins reveals a shift toward more invasive infection contexts, with bloodstream infections (30%) becoming the most represented source. This pattern is consistent across both strain types but shows a differential distribution: cKp remained associated with urinary and bloodstream infections, whereas hvKp exhibited a more systemic profile, with higher representation in bloodstream and respiratory infections. This divergence suggests an early differentiation in clinical behavior between strain types within this period.

The genetic repertoire shows a more structured distribution of virulence determinants compared to 2005–2010. Capsule-associated genes gained prominence, particularly regulators of hypermucoviscosity (14.29%), indicating an increased representation of traits linked to immune evasion and persistence. In parallel, adhesion factors remained relevant, although their relative contribution appears more balanced between type 1 (9.39%) and type 3 fimbriae (15.51%), suggesting diversification within colonization mechanisms rather than simple dominance of a single adhesin type.

Iron acquisition systems showed a notable expansion in diversity rather than frequency alone. Multiple siderophore systems (aerobactin, yersiniabactin, enterobactin, and salmochelin) were detected simultaneously, along with additional iron and heme uptake systems (11.84%). This pattern indicates the emergence of a more complex and redundant iron acquisition network, rather than reliance on isolated mechanisms.

Other virulence components, including secretion systems (T6SS), toxins (colibactin), and metabolic pathways, remained present at lower frequencies but contributed to an increasingly heterogeneous virulence profile. Notably, the detection of metabolic genes (10.61%) suggests an expansion toward traits associated with environmental adaptation and host niche exploitation.

When stratified by strain type, a clear functional divergence becomes evident. In cKp, virulence determinants were distributed across multiple functional groups, with a predominance of adhesion, biofilm formation, and secretion-related factors (80% combined), indicating a more versatile but less specialized profile. In contrast, hvKp showed a strong concentration of detections in capsule and outer envelope-related genes (70%), with a secondary contribution of adhesion factors (25%), reflecting a more focused virulence strategy centered on immune evasion and systemic dissemination.

### 3.4. Genes Reported in the Period 2016–2020

One hundred and sixty studies documented 231 virulence genes (Table 4), representing a substantial increase (76.3%) compared to the previous period. This expansion coincides with a methodological shift, where whole-genome sequencing (47.50%) surpassed conventional PCR (41.88%), suggesting a transition toward more comprehensive detection of virulence repertoires.

Clinical origin showed a more balanced and systemic distribution, with bloodstream infections (24%) as the predominant source, followed by urinary and respiratory tract infections (19% each). Both cKp and hvKp displayed similar distributions across infection sites, indicating a convergence in clinical contexts compared to earlier periods. Demographically, detections were largely concentrated in adults (83%), with lower representation of neonatal and pediatric populations.

At the functional level, a clear reorganization of the virulence repertoire was observed. Iron uptake systems and siderophores became the dominant group, accounting for more than half of all detections (50.56%). This dominance was driven by the simultaneous presence of multiple siderophore systems (aerobactin, yersiniabactin, salmochelin, and enterobactin), indicating a consolidated and redundant network for iron acquisition. In contrast, adhesion factors showed a more moderate contribution, while capsule-associated determinants remained relevant but proportionally less dominant, mainly represented by regulators of hypermucoviscosity.

Other virulence components, including toxins, secretion systems, and metabolic pathways, contributed at lower frequencies, reflecting an increasingly heterogeneous but hierarchically organized virulence profile. Notably, the detection of colibactin and metabolic genes suggests an expansion toward traits associated with host interaction and niche adaptation.

Stratification by strain type revealed a consistent pattern, with iron acquisition systems dominating in both cKp (47.39%) and hvKp (52.39%). However, differences in secondary functional groups persisted: cKp showed a relatively higher contribution of adhesion and biofilm-related genes, whereas hvKp maintained a greater emphasis on capsule and hypermucoviscosity determinants.

Overall, this period is characterized by the consolidation of iron acquisition as the central axis of virulence, accompanied by a more homogeneous clinical distribution and an increasing convergence of virulence profiles between cKp and hvKp, with differences primarily reflected in secondary functional strategies.

### 3.5. Genes Reported in the Period 2021–2025

A total of 474 studies were included, reporting 421 virulence-associated genes (Table 5), representing an 82.3% increase compared to the previous period. This expansion coincides with the predominance of WGS (58.44%), indicating a shift toward more comprehensive genomic approaches and broader detection capacity.

Clinical distribution was relatively consistent across strain types, with bloodstream infections (25%) as the main source, followed by respiratory (21%) and urinary tract infections (20%). Unlike earlier periods, cKp (51%) and hvKp (49%) showed comparable representation and similar anatomical distributions, suggesting a convergence in their clinical occurrence. Both groups were predominantly isolated from adults, although pediatric and neonatal cases remained present at lower proportions.

At the functional level, the virulence repertoire was strongly dominated by iron acquisition systems, which constituted the largest proportion of detections. Siderophores such as yersiniabactin, aerobactin, enterobactin, and salmochelin were consistently represented, with key genes (e.g., *ybtS*, *iucA*, *iutA*, *entB*, *iroB*, *iroN*) recurrently detected, indicating a consolidated role of iron uptake in this period. Capsule-associated determinants also maintained a significant contribution, particularly regulators of hypermucoviscosity (11.92%), with *rmpA* and *rmpA2* accounting for a large fraction of detections.

Adhesion and biofilm-related factors showed a stable contribution, with type 1 and type 3 fimbriae representing similar proportions (~9–10%), and consistent detection of genes such as *fimH* and *mrkD*. Secretion systems, particularly T6SS, displayed moderate representation (4.78%), while other functional groups—including stress response, toxin–antitoxin systems, and metabolic pathways—remained marginal.

Stratification by strain type revealed a largely conserved structure, with iron acquisition systems dominating in both cKp (46%) and hvKp (51%). However, subtle differences persisted: adhesion-related factors were relatively more frequent in cKp, whereas capsule-associated determinants were more prominent in hvKp. Secondary systems such as T6SS showed comparable proportions in both groups, and minor categories contributed minimally to the overall profile.

Overall, this period is characterized by a marked expansion in the detectable virulence repertoire, accompanied by a more balanced distribution between cKp and hvKp and a consolidated predominance of iron acquisition systems as the central component of virulence.

### 3.6. Genes Reported in cKp vs. hvKp

Differences in virulence patterns between cKp and hvKp are reflected in the proportional distribution of functional gene groups. Although both pathotypes share a broad repertoire, their relative contributions vary consistently across categories. Capsule-associated determinants, particularly those related to hypermucoviscosity, show higher representation in hvKp (14.98%) compared to cKp (11.62%).

The most pronounced divergence is observed in iron acquisition systems. While this group predominates in both pathotypes, its relative contribution is higher in hvKp (51.15%) than in cKp (46.04%). Within this category, aerobactin displays the clearest difference, increasing from 11.49% in cKp (726 detections) to 17.13% in hvKp (975 detections). In contrast, yersiniabactin and enterobactin show comparable proportions between both groups, whereas salmochelin remains more frequent in hvKp (9.57%; 521 detections) than in cKp (5.91%; 322 detections).

Conversely, adhesion and biofilm-related factors are more represented in cKp (23.50%) than in hvKp (19.01%), despite their consistent presence in both groups, particularly through type 1 and type 3 fimbriae. These proportional differences indicate a distinct distribution of virulence determinants between pathotypes, with hvKp showing a higher concentration in capsule-associated and iron acquisition functions, while cKp exhibits a relatively greater contribution of adhesion-related factors.

### 3.7. Comparative Analysis of Virulence-Associated Genes Between cKp and hvKp

**Figure 4:**
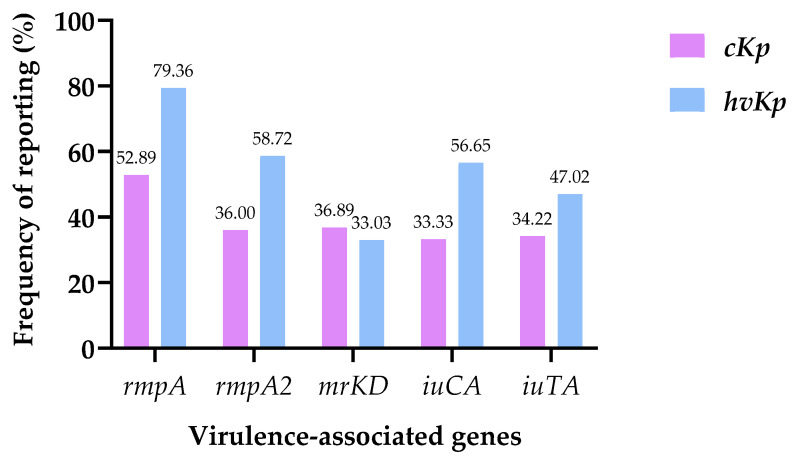
Comparison of selected virulence gene frequencies between cKp and hvKp strains. Bars represent the percentage of studies reporting each virulence-associated gene within classical (cKp) and hypervirulent (hvKp) *K. pneumoniae* subgroups. Frequencies were calculated using the total number of studies reporting each strain type as the denominator.

A total of 886 study-level observations were included in the comparative analysis of virulence-associated genes, corresponding to studies reporting classical *K. pneumoniae* (cKp; n = 450) and hypervirulent *K. pneumoniae* (hvKp; n = 436). Studies without explicit strain classification were excluded from subgroup analyses. Reporting frequencies of selected genes were compared between groups, and effect sizes were estimated as absolute differences in proportions with corresponding 95% confidence intervals (95% CI), while statistical significance was assessed using Fisher’s exact test (Table 6; Figure 4).

Overall, a consistent pattern of differential gene reporting was observed between hvKp and cKp. Genes classically associated with hypervirulence showed significantly higher reporting frequencies among hvKp-related studies than among cKp. Specifically, *rmpA*, *rmpA2*, and *iucA* showed the strongest associations, with absolute differences in reporting frequency exceeding 20 percentage points and highly significant statistical support (*p* < 0.0001 for all comparisons). Among these, *rmpA* showed the largest difference, being reported in 79.36% of hvKp studies compared with 52.89% of cKp studies, corresponding to an absolute difference of 26.47% (95% CI: 20.50–32.73). The gene *iutA* also showed a significantly higher frequency in hvKp, although with a more moderate difference between groups.

In contrast, *mrkD* showed no statistically significant difference between groups, with comparable frequencies in cKp and hvKp studies and no evidence of association with strain classification. The confidence interval for this gene crossed zero, further supporting the absence of a meaningful difference between pathotypes.

Taken together, these findings indicate a heterogeneous distribution of virulence-associated genes between study-defined cKp and hvKp groups. While a subset of genes (*rmpA*, *rmpA2*, *iucA*, and *iutA*) was consistently enriched in hvKp-associated studies, suggesting a closer relationship with the hypervirulent phenotype, other genes such as *mrkD* appeared similarly distributed between groups. This pattern supports a differential distribution of key virulence determinants between the two pathotypes at the study level.

Values are presented as n/N (%), where n represents studies reporting the gene and N represents the total studies in each subgroup. Absolute differences were calculated between hvKp and cKp reporting frequencies. Confidence intervals were estimated using the Newcombe–Wilson method, and statistical significance was determined by Fisher’s exact test.

### 3.8. Influence of Molecular Methods on Virulence Gene Detection

**Figure 5:**
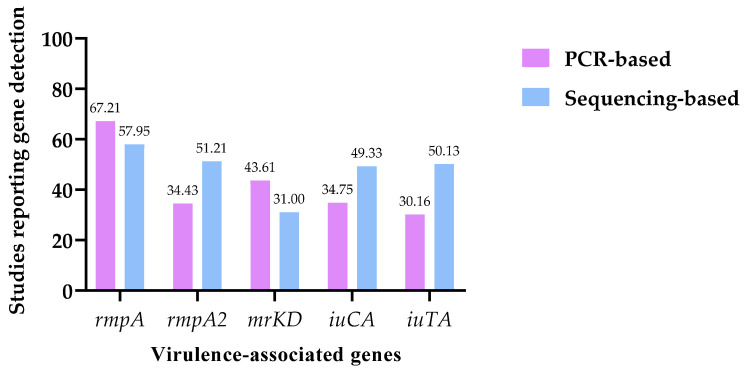
Comparative detection of selected virulence-associated genes by molecular method. Bars represent the percentage of studies reporting each virulence-associated gene within PCR-based and sequencing-based molecular methods. Frequencies were calculated using the total number of studies included in each methodological group as the denominator.

For comparative analysis, the molecular techniques reported across the included studies were grouped into PCR-based methods (n = 305; 45.1%) and sequencing-based methods (n = 371; 54.9%), allowing a standardized assessment of methodological differences in virulence gene reporting (Table 7, Figure 5). The frequency of detection of the five selected virulence-associated genes differed significantly according to the molecular approach used, revealing distinct reporting patterns between methodological groups.

PCR-based methods showed significantly higher reporting frequencies for *rmpA* and *mrkD*. The gene *rmpA* was reported in 67.21% of PCR-based studies compared with 57.95% of sequencing-based studies, corresponding to a positive difference of 9.26% (95% CI: 1.71 to 16.61; *p* = 0.0137). Similarly, *mrkD* was detected in 43.61% of PCR-based studies and 31.00% of sequencing-based studies, with a difference of 12.61% (95% CI: 5.08 to 20.00; *p* = 0.0008).

In contrast, sequencing-based methods showed significantly higher reporting frequencies for several genes commonly associated with hypervirulence. The gene *rmpA2* was identified in 51.21% of sequencing-based studies compared with 34.43% of PCR-based studies, resulting in a negative difference of −16.79% (95% CI: −24.48 to −9.40; *p* < 0.0001). Likewise, the siderophore-associated genes *iucA* and *iutA* were more frequently reported in sequencing-based studies, with differences of −14.58% (95% CI: −22.26 to −7.16; *p* = 0.0002) and −19.97% (95% CI: −27.57 to −12.74; *p* < 0.0001), respectively.

Overall, these findings indicate that the molecular approach used for gene detection may substantially influence the observed distribution of virulence determinants in *K. pneumoniae*. Specifically, PCR-based methods more frequently reported some classical virulence-associated genes, whereas sequencing-based approaches more often identified genes linked to hypervirulence, suggesting that methodological differences should be considered when comparing virulence gene frequencies across studies.

Values are presented as the number of studies reporting each gene relative to the total number of studies included in each methodological group (n/N), with percentages shown in parentheses. PCR-based methods included conventional PCR, multiplex PCR, RT-qPCR, qPCR, RT-PCR, and LAMP, whereas sequencing-based methods included WGS, NGS, mNGS, RNA-seq, and MLST. Differences in proportions were calculated as PCR-based minus sequencing-based frequencies. Positive values indicate higher reporting frequencies in PCR-based studies, whereas negative values indicate higher frequencies in sequencing-based studies. Confidence intervals were estimated using the Newcombe–Wilson method with continuity correction, and statistical significance was assessed using Fisher’s exact test.

### 3.9. Regional Analysis of K. pneumoniae Virulence Genes

**Figure 6:**
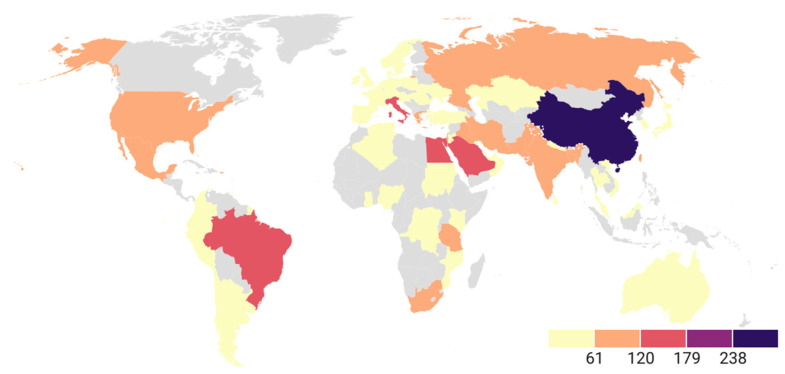
Choropleth map of the number of detections of *K. pneumoniae* virulence genes identified by country (2005–2025). Asia has the highest burden, followed by Europe and America; Africa shows a more homogeneous distribution and Oceania has fewer reports.

Regional analysis of *K. pneumoniae* virulence genes reveals heterogeneous patterns that reflect differences in geographic distribution, (Figure 6). Asia is the region that contributes the largest volume of detections, both in terms of the number of genes (376) and the number of detections (5859); here, the repertoire oriented towards iron uptake systems and capsule regulation clearly predominates. The siderophores aerobactin (15.70%) and yersiniabactin (15.57%) are the most prominent components, accompanied by salmochelin (8.19%) and enterobactin (8.82%), reinforcing iron acquisition as a key factor in the virulence of this pathogen in this region. This is further supported by the high representation of hypermucoviscosity-regulating genes (12.95%), along with type 1 (7.63%) and type 3 (9.83%) fimbriae. Overall, Asia exhibits a profile characterized by a strong combination of hypercapsule, highly efficient siderophores, and adhesins.

On the other hand, Europe shows a distribution less proportional to hypervirulence, but siderophores remain the main functional group. Yersiniabactin reaches its highest value among all regions here (17.86%), accompanied by aerobactin (10.38%) and enterobactin (9.47%), while the contribution of salmochelin (4.66%) is lower compared to Asia. Capsule genes and hypermucoviscosity regulation also show a considerable presence (9.69%). On the other hand, in this region there is a relevant proportion of detections associated with type 3 fimbriae (13.28%), and to a lesser extent type 1 fimbriae (9.47%).

In the Americas, iron uptake systems are also predominant, although factors such as aerobactin (10.20%), enterobactin (6.23%), and salmochelin (5.43%) are less prevalent. Yersiniabactin again stands out as the most represented siderophore (15.23%). Capsule regulation and hypermucoviscosity also maintain a considerable value (10.60%), and adhesins show significant levels in both type 1 (8.21%) and type 3 (10.73%) fimbriae.

In Africa, siderophores remain the most frequently detected genes; enterobactin, yersiniabactin, and type 1 fimbriae reach identical values (12.93%). Aerobactin shows moderate values (8.62%), while hypermucoviscosity presents a relatively low percentage compared to regions such as the Americas and Asia (8.86%). The presence of T6SS (7.43%) is higher than in other regions. The African profile is characterized by a more homogeneous distribution, without a dominant functional group.

Finally, Oceania shows a limited pattern of available detections. In this region, high proportions of hypermucoviscosity (25.00%) and type 3 fimbriae (33.33%) are observed, whereas the contribution of siderophores is low and mainly restricted to aerobactin (8.33%) and a single detection of yersiniabactin (4.17%). Other functional groups appear at low proportions or are absent. Despite this, the prominent presence of adhesins and capsular regulators suggests that isolates from this region may be associated with colonization patterns and hypermucoviscosity.

### 3.10. Results of Risk of Bias and Methodological Quality Assessment

The EMMO-Vir tool was applied to all 676 included studies to assess methodological quality and potential sources of bias. The overall mean quality score was 84.14%, corresponding to a moderate level according to the EMMO-Vir classification (0–65% low, 66–85% moderate, 86–100% high). Across domains, mean scores were 81.45% for Domain 1 (Methodological Quality), 81.46% for Domain 2 (Technical Quality), 95.01% for Domain 3 (Methodology and Bias), and 88.17% for Domain 4 (Interpretation and Transparency). These values represent aggregated estimates and should be interpreted with caution, as variability between individual studies may not be fully captured. The most frequent deficiencies were related to the lack of replication or reproducibility measures (Item 2.8, 35.19%), absence of positive and negative controls (Item 2.6, 42.40%), and limited cross-validation using alternative techniques (Item 2.7, 65.98%). Several items showed high levels of compliance (≥95%), including consistency between interpretation and molecular techniques (Item 4.1, 99.70%), use of standardized experimental conditions (Item 3.2, 98.37%), clear description of molecular methods (Item 2.1, 98.67%), comprehensive reporting of results (Item 3.4, 97.49%), and description of known or potential gene functions (Item 4.3, 97.49%), although this may reflect reporting practices rather than true methodological rigor. No study was excluded solely based on quality assessment; however, quality ratings were considered during the interpretation of findings.

Funnel plots were not constructed due to the heterogeneity of study designs, outcomes, and reporting methods, which limited the applicability of quantitative bias assessment. No formal statistical assessment of publication bias was performed; therefore, the presence of publication bias cannot be ruled out.

### 3.11. Certainty of Evidence

The certainty of the evidence was assessed narratively using predefined domains, including consistency, methodological quality, reporting completeness, and biological plausibility. Regarding consistency, similar patterns were observed across studies, including the predominance of iron acquisition systems and siderophores across the evaluated periods, as well as differences between classical (cKp) and hypervirulent (hvKp) strains in terms of adhesion and capsular determinants. In terms of methodological quality, the mean EMMO-Vir score was 84.18%, corresponding to a moderate level. Lower scores were observed in reproducibility (35.19%) and the use of controls (42.40%). Regarding reporting completeness, most studies (≥94%) reported the genes analyzed, molecular techniques, and origin of isolates. Cross-validation using alternative methods was reported in 65.98% of studies. Biological plausibility was evaluated based on the alignment between reported findings and known functional roles of virulence genes. Overall, the certainty of the evidence was considered moderate for the main findings and lower for more specific observations. Inconsistency could not be formally assessed due to the absence of quantitative synthesis.

## 4. Discussion

This systematic review, which consolidates data from 676 studies published between 2005 and 2025, demonstrates a sustained expansion in the reporting of knowledge regarding the virulence genetics of *K. pneumoniae*, both in the volume of publications and in the diversity of genes described. Taken together, these findings support a model in which *K. pneumoniae* pathogenicity is organized by functional modules (iron uptake, capsule, hypermucoviscosity, adhesion, biofilms, secretion systems, and toxins), although it is important to clarify that these patterns reflect reported detections in the literature rather than direct measures of prevalence or biological distribution. Notably, a significant proportion of the evidence is concentrated in a small core of genes that are repeatedly reported across the included studies. To the best of our knowledge, this is the first systematic review focused on the exhaustive synthesis of virulence-associated genes reported in *K. pneumoniae*. Our findings provide a comprehensive overview of the temporal increase in the identification of reported virulence-associated genes, largely driven by advances in molecular techniques rather than necessarily reflecting true biological evolution, but instead improvements in detection capacity that have been crucial in streamlining research in this field.

Studies have demonstrated that plasmids can mediate the dissemination of virulence genes, resulting in the emergence of hvKp, or even hypervirulent and drug-resistant *K. pneumoniae* [5,19,20]. Siderophore-associated genes such as *iucA* (aerobactin) and iroB (in this context, the pattern observed in our results—from 37 genes reported between 2005 and 2010 to 421 genes between 2021 and 2025) are better explained by improvements in detection capacity and methodological advances rather than by a direct evolutionary shift. The shift from traditional phenotypic and targeted molecular approaches toward genome-based and gene-centric strategies has improved the characterization of virulence and resistance determinants, particularly those associated with mobile genetic elements such as plasmids. This paradigm shift—from species-level identification to gene-level resolution—is increasingly recognized as central to modern clinical microbiology and surveillance efforts [21]. Therefore, the trends observed in this review primarily reflect reporting frequency and detection patterns, as well as the technical capacity of studies to capture virulence determinants.

Across the included studies, and consistent with this framework, a cross-cutting finding is that genes associated with iron and heme uptake dominate reported detections. This aligns with previous findings [22], which indicate that siderophore production is a predominant strategy among numerous pathogens, including *K. pneumoniae*, and that the ability to synthesize multiple siderophores may facilitate colonization across different tissues by compensating for host-mediated neutralization mechanisms.

Notably, in the human body, free iron concentrations are extremely low (~10^−18^ M) due to tightly regulated sequestration and transport systems, with more than 80% of total iron confined within heme-containing proteins such as hemoglobin, myoglobin, cytochromes, and iron-dependent enzymes. This restriction is further enhanced during infection, leading to reduced transferrin saturation and limited iron availability; in this context, the efficiency of bacterial iron acquisition may influence the transition from colonization to infection [23]. Siderophore-associated genes such as *iucA* (aerobactin) and *iroB* (salmochelin) have demonstrated high accuracy for identifying hypervirulent strains and are strongly associated with increased virulence [24]. Our results are consistent with this interpretation, as genes involved in iron and heme acquisition are repeatedly identified, highlighting their prominence as frequently reported determinants. However, their detection alone does not necessarily imply clinical virulence.

In the second-most frequently detected functional group, genes related to hypermucoviscosity and capsule regulation were prominent. *rmpA*, *rmpA2*, and *magA* are known to enhance capsule production, whereas *peg-344* has been consistently reported as a highly accurate biomarker of hypervirulent *K. pneumoniae*, despite not being directly involved in capsule synthesis [24]. In hvKp, the presence of genes such as *rmpA* and *rmpA2* is often accompanied by iron acquisition determinants, particularly the siderophore systems aerobactin (iuc genes) and salmochelin (iro genes), suggesting a profile oriented toward maximizing survival and growth within the host. These factors together with increased capsule production confer greater invasive capacity and metastatic dissemination potential to hvKp, in contrast to classical *K. pneumoniae* (cKp), which is primarily associated with opportunistic nosocomial infections in patients with comorbidities or immunocompromised conditions [25]. An important implication of these findings is that, although a total of 474 virulence-associated genes were documented, detections are concentrated within a relatively small subset that accounts for a large proportion of the records, exemplified by *mrkD*, *iutA*, *iucA*, *rmpA2*, and *rmpA*, each reported in more than 200 studies included in this review. This pattern suggests that these loci may represent a set of consistently reported and clinically relevant virulence determinants in *K. pneumoniae*, which is why many genomic surveillance studies prioritize this subset for detection panels and epidemiological surveillance [26].

Genes associated with secretion systems, particularly the type VI secretion system (T6SS), have been increasingly recognized due to their role in multiple processes related to bacterial fitness, including interbacterial competition, adhesion, invasion, and host colonization [27]. In this context [28] analyzed virulence genes in T6SS-positive clinical isolates of *K. pneumoniae* and observed that, compared to T6SS-negative strains, T6SS-positive isolates exhibited a higher frequency of virulence genes, including *rmpA*, *fimH*, *entB*, *kfu*, and *ybtS*. Taken together, these findings are consistent with our results, as most detections of T6SS-associated genes were concentrated in more recent periods. This temporal pattern likely reflects the consolidation of high-resolution genomic techniques, such as WGS and NGS, which have expanded the identification of virulence loci and complete secretion systems in bacterial genomes. In addition, the recognized role of the type VI secretion system in pathogenesis and microbial competition has contributed to the growing research interest in this system in recent years. This study highlights that, with the advent of whole-genome sequencing, complete sets of type II secretion system (T2SS) genes have been identified across a wide range of Gram-negative pathogens [29]. Rather than representing a recent discovery, these findings reflect the expansion of genomic approaches, which have enabled a more comprehensive characterization of T2SS distribution and genetic composition. This secretion system plays a key role in bacterial adaptation and virulence. Recently [11] described that the type II secretion system (T2SS) is involved in the export of folded proteins to the extracellular environment, contributing to bacterial virulence by transporting enzymes and factors that facilitate host colonization and tissue interaction.

Furthermore, biofilm formation is an important virulence trait of *K. pneumoniae*. Fimbriae promote adhesion to non-biological surfaces, thereby facilitating catheter-related infections. Type 1 and type 3 fimbriae are encoded by the *fim* and *mrk* gene clusters, respectively. In particular, type 1 fimbriae are key contributors to urinary tract infections and exhibit a high affinity for mannose residues on bladder epithelial cells [30].

When comparing the detection rates of type 1 fimbriae proportionally to the total number of virulence genes reported in each clinical source, a higher frequency was observed in studies associated with the urinary tract (9.85%) compared to other sources such as blood (8.48%) and the respiratory tract (8.30%). Furthermore, it has been reported that type 3 fimbriae play a more significant role in biofilm formation than type 1 fimbriae [30].

Finally, genes encoding genotoxic toxins, such as colibactin, were detected. Colibactin has recently been considered a novel virulence factor and has attracted increasing attention. This bacterial secondary metabolite, encoded by the *pks* island, can interfere with the cell cycle in eukaryotic cells and cause cellular damage; consequently, colibactin production may enhance bacterial virulence and pathogenicity [31]. When comparing colibactin detections proportionally across different clinical sources, this genotoxin showed the highest relative frequency in studies associated with the nervous system (5.37%), followed by blood (3.77%), whereas the urinary and respiratory tracts exhibited identical and lower proportions (3.48%). This pattern is consistent with experimental evidence suggesting a role of the *pks* locus in invasive disease, particularly in central nervous system infections. In murine models, hypervirulent *K. pneumoniae* strains carrying the *pks* island have been shown to induce meningitis and display a marked meningeal tropism Nevertheless, the clinical implications of this distribution remain uncertain and require further investigation [32].

Regarding regional distribution, Asia contributed the largest volume in both diversity (376 genes) and number of detections (5859), with a profile characterized by a high prevalence of siderophore-associated genes, including aerobactin (*iutA*, *iucA*, *iucB*, *iucC*) and yersiniabactin (*ybtS*, *irp1*, *irp2*, *ybt*, *ybtQ*), as well as regulators linked to hypermucoviscosity (*rmpA*, *rmpA2*, *magA*, *peg-344*). These results are consistent with previous reports describing a higher prevalence of hypervirulent *K. pneumoniae* lineages in the Asian Pacific Rim, commonly associated with virulence plasmid-encoded factors such as aerobactin and increased capsule production [25]. Furthermore, invasive syndrome caused by hvKp was initially described in Asia and later recognized globally; consequently, early reports have been largely concentrated in Asian countries, particularly China and Taiwan [33]. However, this pattern may also reflect differences in research intensity and reporting rather than the true epidemiological distribution.

This summary should be interpreted considering methodological heterogeneity among studies and detection bias depending on the technique used. In addition, the described pattern summarizes detections reported by the included studies and should not be interpreted as direct estimates of prevalence or pathogenic potential. Additionally, the lack of consistent clinical metadata (e.g., colonization vs. infection, ICU vs. non-ICU, or host condition) limits the ability to establish associations between gene detection and clinical outcomes. Even with these limitations, the consistency of the iron uptake domain, the co-occurrence with capsule and hypermucoviscosity, and the concentration of determinants in a reduced core support its value as a priority axis for molecular surveillance and comparative studies. Molecular research needs to continue advancing to elucidate and further expand knowledge about the pathogenicity mechanisms of this globally relevant pathogen.

## 5. Conclusions

To our knowledge, this is the first systematic review focused on the identification and reporting patterns of virulence-associated genes in *K. pneumoniae* over the past two decades. This study documents the variability and frequency of reported virulence genes, as well as the molecular techniques used for their detection.

Based on twenty years of evidence, our findings suggest that the virulence repertoire of *K. pneumoniae*, as described in the literature, is structured into functional modules and recurrent determinants. The apparent expansion of the reported genetic repertoire likely reflects both increased surveillance efforts and the progressive adoption of higher-resolution molecular techniques capable of capturing complete loci and patterns of co-occurrence. Therefore, the results should be interpreted as reported detections rather than direct estimates of true prevalence or biological evolution.

In this context, iron acquisition systems consistently emerge as central components associated with clinical pathogenicity, representing a recurring theme across different clinical settings. Although a total of 474 genes were identified across the studies, the evidence was concentrated in a limited subset of determinants—such as *mrkD*, *iutA*, *iucA*, *rmpA2*, and *rmpA*—which accounted for a disproportionately high proportion of detections despite representing a small fraction of the total genes described. This pattern highlights a core set of frequently reported determinants with potential biological and epidemiological relevance.

Taken together, these findings support prioritizing a subset of virulence-associated genes as candidate markers for genomic surveillance, particularly in hospital settings where virulence and antimicrobial resistance converge. Diagnostic and monitoring strategies targeting these genes could capture a substantial proportion of the clinically relevant virulence potential of *K. pneumoniae*. However, the findings also underscore the need for further research to standardize molecular detection methods, improve reporting consistency, and integrate clinical metadata in order to better understand the relationship between gene presence and expression and clinical outcomes.

## Figures and Tables

**Table 1:** Temporal trends in the reporting of virulence genes and predominant detection methods (2005–2025).

	2005–2010	2011–2015	2016–2020	2021–2025
Studies included	8	34	160	474
Total isolates	416	2.803	13.561	52.387
Distinct genes detected	37	131	231	421
Detection records	42	169	2.197	6.672
Distribution: strain type	cKp 54% hvKp 46%	cKp 65% hvKp 35%	cKp 53% hvKp 47%	cKp 54% hvKp 46%
Dominant technique	PCR (100%)	PCR (67.65%) WGS (17.65%)	WGS (47.50%) PCR (41.88%)	WGS (58.44%) PCR (32.49%)

Abbreviations: cKp, classical *Klebsiella pneumoniae*; hvKp, hypervirulent *Klebsiella pneumoniae*; PCR, polymerase chain reaction; WGS, whole-genome sequencing.

**Table 2:** Virulence genes of *K. pneumoniae* recorded in the period 2005–2010, according to functional group.

Functional Group/Virulence Factors	N° of Genes	%	Genes
Structural genes of the K locus	3	7.1%	*wza*, *wzc*, *wzy*
Capsular sugar synthesis	2	7.1%	*gnd*, *uge*
Capsular serotype genes	1	2.4%	*wzi*
Capsule regulation and hypermucoviscosity (hvKp)	4	19.0%	*rmpA*, *magA*, *rcsB*, *peg-344*
LPS/O-antigen/lipid A	2	4.8%	*wabG*, *wzx*
Lipid A and charge modification	0	0.0%	*_*
Siderophores and iron uptake Aerobactin	0	0.0%	*_*
Siderophores and iron uptake Enterobactin	1	2.4%	*fepA*
Siderophores and iron uptake Yersiniabactin	1	2.4%	*fyuA*
Siderophores and iron uptake Salmochelin	0	0.0%	*_*
Other iron and heme uptake systems	1	2.4%	*kfu*
Fimbriae and adhesins Type 1 fimbriae	9	21.4%	*fimA*, *fimB*, *fimC*, *fimD*, *fimE* *fimF*, *fimG*, *fimI*, *fimK*
Fimbriae and adhesins Type 3 fimbriae	5	11.9%	*mrkA*, *mrkB*, *mrkC*, *mrkD*, *mrkF*
Other fimbrial operons and pili	0	0.0%	*_*
Invasive/iron-regulated adhesins	0	0.0%	*_*
Matrix factors and biofilm regulation	0	0.0%	*_*
Type VI secretion system (T6SS)	7	16.7%	*impG*, *impH*, *impJ*, *icmF/tssM* *dotU/tssL*, *tli*, *impF*
Type II secretion system (T2SS)	0	0.0%	*_*
Toxins and genotoxins colibactin	0	0.0%	*_*
Other toxins and cytotoxic factors	0	0.0%	*_*
Regulators, stress response and outer membrane	1	2.4%	*ompA*
Toxin–antitoxin systems and persistence	0	0.0%	*_*
Nutrient acquisition and metabolism (excluding iron)	0	0.0%	*_*
Allantoin, purineand glycosylate metabolism	0	0.0%	*_*

Abbreviations: LPS, lipopolysaccharide; hvKp, hypervirulent *Klebsiella pneumoniae*. The number (No.) reflects the number of included studies in which at least one gene from each functional group was reported. Percentages (%) indicate the relative frequency of reporting of each functional group across the included studies. Gene names include canonical genes as well as reported variants or homologs, as described in the original studies.

**Table 3:** Virulence genes of *K. pneumoniae* recorded in the period 2011–2015, according to functional group.

Functional Group/Virulence Factors	No. of Genes	%	Genes
Structural genes of the K locus	6	2.86%	*wzc*, *wzb*, *wzy*, *wcaG*, *wcuF*, *wcoU*
Capsular sugar synthesis	3	1.63%	*manB*, *uge*, *ugeE*
Capsular serotype genes	1	1.22%	*wzi*
Capsule regulation and hypermucoviscosity (hvKp)	10	14.29%	*rmpA*, *rmpA2*, *c-rmpA*, *magA*, *peg-344* *orf2*, *glf*, *rcsB*, *ibrB*, *peg-589*
LPS/O-antigen/lipid A	1	2.86%	*wabG*
Lipid A and charge modification	0	0.00%	*_*
Siderophores and iron uptake Aerobactin	8	6.53%	*iuc*, *iucA*, *iucB*, *iucC*, *iucD*, *iut*, *iutA*, *aero*
Siderophores and iron uptake Enterobactin	3	1.63%	*ent*, *entB*, *fepA*
Siderophores and iron uptake Yersiniabactin	7	6.53%	*ybt*, *ybtS*, *irp1*, *irp2*, *ybt9*, *fyu*, *fyuA*
Siderophores and iron uptake Salmochelin	5	4.08%	*iro*, *iroB*, *iroC*, *iroD*, *iroN*
Other iron and heme uptake systems	20	11.84%	*fec*, *fecA*, *fecI*, *fecR*, *fhuA*, *fhuB* *fhuC*, *fhuD*, *hmuR*, *hmuS*, *hmuT*, *hmuV* *hmuU*, *kfu*, *kfuB*, *kfuC*, *sitA*, *sitB*, *sitC*, *sitD*
Fimbriae and adhesins Type 1 fimbriae	12	9.39%	*fim*, *fimA*, *fimB*, *fimC*, *fimD*, *fimE* *fimF*, *fimG*, *fimH*, *fimI*, *fimK*, *fimH-1*
Fimbriae and adhesins Type 3 fimbriae	9	15.51%	*mrk*, *mrkA*, *mrkB*, *mrkC*, *mrkD* *mrkF*, *mrkH*, *mrkI*, *mrkJ*
Other fimbrial operons and pili	13	5.31%	*kpa*, *kpb*, *kpd*, *kpe*, *kpf*, *kpg*, *ecp* *ecpA*, *ecpB*, *ecpC*, *ecpD*, *ecpE*, *ecpR*
Invasive/iron-regulated adhesins	0	0.00%	*_*
Matrix factors and biofilm regulation	0	0.00%	*_*
Type VI secretion system (T6SS)	2	0.82%	*vgrG/tssI*, *hcp/tssD*
Type II secretion system (T2SS)	0	0.00%	*_*
Toxins and genotoxins colibactin	3	1.63%	*clb*, *clb2*, *clbR*
Other toxins and cytotoxic factors	2	0.82%	*khe*, *hlyA*
Regulators, stress response and outer membrane	3	1.63%	*ompA*, *pagO*, *traT*
Toxin–antitoxin system and persistence	0	0.00%	*_*
Nutrient acquisition and metabolism (excluding iron)	1	0.82%	*ureA*
Allantoin, purine and glycosylate metabolism	22	10.61%	*allA*, *allB*, *allC*, *allD*, *allR*, *allS*, *ybbW* *ybbY*, *ybbA*, *ylbE*, *ybbP*, *ybbQ*, *ybbS*, *ybbT* *ybbU*, *ybbX*, *glxK*, *glxR*, *fdrA*, *glc*, *hyi*, *ylbF*

Abbreviations: LPS, lipopolysaccharide; hvKp, hypervirulent *Klebsiella pneumoniae*. The number (No.) reflects the number of included studies in which at least one gene from each functional group was reported. Percentages (%) indicate the relative frequency of reporting of each functional group across the included studies. Gene names include canonical genes as well as reported variants or homologs, as described in the original studies.

**Table 4:** Virulence genes of *K. pneumoniae* recorded in the period 2016–2020, according to functional group.

Functional Group/Virulence Factors	No. of Genes	%	Genes
Structural genes of the K locus	12	1.88%	*wza*, *wzc*, *wzm*, *wzb*, *wzc50*, *wzy* *cps*, *cpsA*, *cpsB*, *wcaG*, *wcaJ*, *wcbF*
Capsular sugar synthesis	7	1.17%	*gmd*, *manB*, *falF*, *ugd*, *rmlB*, *uge*, *wbaP*
Capsular serotype genes	2	0.51%	*wzi*, *wzi-705*
Capsule regulation and hypermucoviscosity (hvKp)	12	11.19%	*wmpA*, *wmpA2*, *p-rmpA*, *p-rmpA2*, *c-rmpA* *rmpC*, *magA*, *orf10*, *kvgS*, *rcsB*, *mviM*, *peg-344*
LPS/O-antigen/lipid A	7	1.58%	*waaE*, *wabG*, *wabN*, *wbbM*, *ofr*, *rfaH*, *wzx*
Lipid A and charge modification	0	0.00%	*_*
Siderophores and iron uptake Aerobactin	11	13.48%	*iuc*, *iucA*, *iucB*, *iucC*, *iucD* *iuc1*, *iut*, *iutA*, *iutB*, *iutC*, *aero*
Siderophores and iron uptake Enterobactin	20	8.09%	*ent*, *entA*, *entB*, *entC*, *entD*, *entE*, *entF* *entH*, *entS*, *fep*, *fepB*, *fepC*, *fepD*, *fepG* *fes*, *febI*, *febR*, *febD*, *febC*, *fepA*
Siderophores and iron uptake Yersiniabactin	30	16.33%	*ybt*, *ybtA*, *ybtB*, *ybtE*, *ybtO*, *ybtQ*, *ybtS*, *ybtT* *ybtU*, *ybuD*, *ybtL*, *ybtX*, *irp*, *irp1*, *irp2* *irp3*, *irp5*, *ybt0*, *ybt1*, *ybt4*, *ybt9*, *ybt10* *ybt14*, *ybt16*, *ybt17*, *Airp1*, *Airp2*, *fyu*, *fyuA*, *ybtR*,
Siderophores and iron uptake Salmochelin	8	7.58%	*iro*, *iro1*, *iro2*, *iroB*, *iroC*, *iroD*, *iroE*, *iroN*
Other iron and heme uptake systems	11	5.09%	*fec*, *fecA*, *fecB*, *fecI*, *fecR* *ybdA*, *kfu*, *kfuA*, *kfuB*, *kfuC*, *kfuD*
Fimbriae and adhesins Type 1 fimbriae	12	6.31%	*fim*, *fimA*, *fimB*, *fimC*, *fimD*, *fimE* *fimF*, *fimG*, *fimH*, *fimI*, *fimK*, *fimH-1*
Fimbriae and adhesins Type 3 fimbriae	10	13.17%	*mrk*, *mrkA*, *mrkB*, *mrkC*, *mrkD* *mrkF*, *mrkH*, *mrkI*, *mrkJ*, *mrkK*
Other fimbrial operons and pili	8	1.98%	*kpn*, *ecpA*, *ecpB*, *ecpC*, *ecpD*, *ecpE*, *ecpR*, *pilW*
Invasive/iron-regulated adhesins	0	0.00%	*_*
Matrix factors and biofilm regulation	4	0.25%	*pgaA*, *pgaB*, *pgaC*, *bcsA*
Type VI secretion system (T6SS)	8	0.46%	*impG*, *impH*, *impJ*, *vasG* *icmF/tssM*, *dotU/tssL*, *impF*, *pld1*
Type II secretion system (T2SS)	10	0.51%	*gspE*, *gspG*, *pulB*, *pulC*, *pulD* *pulE*, *pulG*, *pulO*, *pulS*, *PulN*
Toxins and genotoxins: colibactin	22	5.80%	*clb*, *clb2*, *clb3*, *clb5*, *clbA*, *clbB*, *clbC* *clbD*, *clbE*, *clbF*, *clbG*, *clbH*, *clbI*, *clbJ*, *clbK* *clbL*, *clbM*, *clbN*, *clbO*, *clbP*, *clbQ*, *clbR*
Other toxins and cytotoxic factors	5	0.36%	*hly*, *CNF-1*, *khe*, *vatD*, *cnf*
Regulators, stress response and outer membrane	5	0.81%	*ompA*, *pagO*, *traT*, *shiF*, *htrA*
Toxin–antitoxin systems and persistence	8	0.41%	*hipA*, *hipB*, *vapB*, *vapC*, *hpd*, *doc*, *mazE*, *mazF*
Nutrient acquisition and metabolism (excluding iron)	3	0.66%	*ureA*, *ureE*, *ureD*
Allantoin, purine and glycosylate metabolism	16	2.39%	*allA*, *allB*, *allC*, *allD*, *allR*, *allS*, *ybbW* *ybbY*, *ylbE*, *glxK*, *glxR*, *fdrA*, *gcl*, *hyi*, *ylbF*, *arcC*

Abbreviations: LPS, lipopolysaccharide; hvKp, hypervirulent *Klebsiella pneumoniae*. The number (No.) reflects the number of included studies in which at least one gene from each functional group was reported. Percentages (%) indicate the relative frequency of reporting of each functional group across the included studies. Gene names include canonical genes as well as reported variants or homologs, as described in the original studies.

**Table 5:** Virulence genes of *K. pneumoniae* recorded in the period 2021–2025, according to functional group.

Functional Group/Virulence Factors	No. of Genes	%	Genes
Structural genes of the K locus	30	1.81%	*wza*, *wzc*, *wzm*, *wzb*, *wzc50*, *wzxE*, *wzt*, *wzy* *cps*, *cpsA*, *cpsB*, *kps*, *KpsI*, *kpsM*, *KpsF*, *wcah* *wcai*, *wcaM*, *wcaG*, *wcaJ*, *wceN*, *wceM*, *wacJ*, *wckI* *wcqC*, *wcsF*, *wcsR*, *wcuT*, *wcoV*, *wcoU.*
Capsular sugar synthesis	23	1.48%	*gmd*, *manC*, *manB*, *gnd*, *galF*, *galU*, *ugd*, *rmlB* *rmlC*, *rmlD*, *galA*, *galB*, *galC*, *galD*, *galE*, *galM* *galP*, *galR*, *galS*, *uge*, *ugeF*, *gmhA*, *wbaP.*
Capsular serotype genes	4	0.49%	*wzi*, *wzi1*, *wzi23*, *wziK24*.
Capsule regulation and hypermucoviscosity (hvKp)	21	11.92%	*rmpA*, *rmpA1*, *rmpA2*, *rmpA3*, *p-rmpA*, *p-rmpA2*, *c-rmpA* *rmpB*, *rmpC*, *rmpD*, *magA*, *kvrA*, *kvrB*, *kvgA*, *kvgS*, *rcs* *rcsB*, *capL*, *peg-344*, *peg-589*, *peg-1631*.
LPS/O-antigen/lipid A	17	1.08%	*wabG*, *wabH*, *wabN*, *waaU*, *wbbO*, *wbbM*, *wbbN*, *wbbY* *rfaD*, *rfaE*, *rfaF*, *rfaQ*, *wecA*, *wzx*, *acpXL*, *pagP*, *wzzB*.
Lipid A and charge modification	9	0.15%	*lpx*, *lpxA*, *lpxB*, *lpxC*, *lpxD*, *arnD*, *kds*, *kdsA*, *kdsB*.
Siderophores and iron uptake Aerobactin	18	14.15%	*iuc*, *iucA*, *iucB*, *iucC*, *iucD*, *iucE*, *iucF*, *iuc19* *iuc1*, *iuc2*, *iuc3*, *iuc4*, *iuc5*, *iut*, *iutA*, *iutB*, *Kvar_1549*, *aero*.
Siderophores and iron uptake Enterobactin	25	9.70%	*ent*, *entA*, *entB*, *entB1*, *entB2*, *entC*, *entD*, *entE* *entF*, *entH*, *entS*, *fep*, *fepB*, *fepC*, *fepD* *fepG*, *fes*, *fesE*, *fesF*, *febA*, *febB*, *febF*, *febD*, *febC*, *fepA*.
Siderophores and iron uptake Yersiniabactin	30	15.53%	*ybt*, *ybtA*, *ybtB*, *ybtE*, *ybtP*, *ybtQ*, *ybtS*, *ybtT* *ybtU*, *ybtD*, *ybtL*, *ybtX*, *irp*, *irp1*, *irp2* *ybt1*, *ybt2*, *ybt9*, *ybt10*, *ybt12*, *ybt13*, *ybt14* *ybt15*, *ybt16*, *ybt17*, *ybt19*, *Airp1*, *Airp2*, *fyu*, *fyuA*.
Siderophores and iron uptake Salmochelin	8	7.10%	*iro*, *iro1*, *iro2*, *iroB*, *iroC*, *iroD*, *iroE*, *iroN*.
Other iron and heme uptake systems	31	2.28%	*tonB*, *fecA*, *fecI*, *fecR*, *fur*, *feo*, *ybdA*, *ybdC* *ybdG*, *ybdK*, *ybdL*, *ybdM*, *ybdO*, *ybdZ*, *feoA*, *feoB* *feoC*, *fhuD*, *cirA*, *kfu*, *kfuA*, *kfuB*, *kfuC*, *kfuD* *sitA*, *sitB*, *sitC*, *sitD*, *ChuA*, *ChuS*, *ChuU*.
Fimbriae and adhesins Type 1 fimbriae	14	9.05%	*fim*, *fimA*, *fimB*, *fimC*, *fimD*, *fimE*, *fimF* *fimG*, *fimH*, *fimM*, *fimI*, *fimJ*, *fimK*, *fimH-1*.
Fimbriae and adhesins Type 3 fimbriae	12	9.82%	*mrk*, *mrkA*, *mrkB*, *mrkC*, *mrkD*, *mrkF* *mrkH*, *mrkI*, *mrkJ*, *mrkL*, *mrkK*, *mrkM*.
Other fimbrial operons and pili	25	2.53%	*kpn*, *stbA*, *stbB*, *stbC*, *stbD*, *stbE*, *kpiA*, *kpiB* *kpiC*, *kpiD*, *kpiE*, *kpiF*, *kpiG*, *ecp*, *ecpA*, *ecpB*, *ecpC* *ecpD*, *ecpE*, *ecpR*, *pilR*, *pilT*, *pilD*, *pilW*, *pilU*.
Invasive/iron-regulated adhesins	15	0.24%	*iha*, *air*, *ibeB*, *afa*, *papE*, *papF*, *papC* *papD*, *papX*, *focA*, *focC*, *focD*, *sfaD*, *sfaE*, *dfaF*.
Matrix factors and biofilm regulation	7	0.15%	*pgaA*, *pgaB*, *pgaC*, *pgaD*, *agn43*, *bssS*, *clpK*.
Type VI secretion system (T6SS)	35	4.78%	*tssA*, *tssB*, *tssC*, *tssD*, *tssE*, *tssF*, *tssG*, *tssH* *tssI*, *tssJ*, *tssK*, *tssL*, *tssM*, *imp*, *impG*, *impH*, *impJ* *vasG*, *icmF/tssM*, *dotU/tssL*, *vasE/tssK*, *clpV/tssH*, *vgrG/tssI* *hcp/tssD*, *impA/tssD*, *vasA/impG*, *vipA/tssB* *vipB/tssC*, *sciN/tssJ*, *tli1*, *tle1*, *sci*, *dotU*, *impF*, *impN*.
Type II secretion system (T2SS)	25	0.45%	*gspC*, *gspD*, *gspE*, *gspG*, *gspH*, *gspI*, *gspJ*, *gspL* *gspM*, *gspN*, *pulB*, *pulC*, *pulD*, *pulE*, *pulF*, *pulG* *pulH*, *pulI*, *pulJ*, *pulK*, *pulL*, *pulO*, *pulP*, *pulS*, *PulN*.
Toxins and genotoxins colibactin	23	3.37%	*clb*, *clb1*, *clb2*, *clb3*, *clbA*, *clbB*, *clbC*, *clbD* *clbE*, *clbF*, *clbG*, *clbH*, *clbI*, *clbJ*, *clbK*, *clbL* *clbM*, *clbN*, *clbO*, *clbP*, *clpQ*, *clbR*, *clbS*.
Other toxins and cytotoxic factors	9	0.18%	*hlyA*, *EAST-1*, *CNF-1*, *khe*, *SAT*, *usp*, *astA*, *hlyE*, *VAT*.
Regulators, stress response and outer membrane	12	0.84%	*OmpA*, *pagO*, *ompX*, *ompR*, *ompW*, *ompN* *ompC*, *traT*, *shiF*, *lon*, *htrA*, *zapA*.
Toxin–antitoxin systems and persistence	5	0.07%	*hipA*, *hipB*, *phd*, *doc*, *ratA*.
Nutrient acquisition and metabolism (excluding iron)	7	0.81%	*ureC*, *ureD*, *ureE*, *ureF*, *ureG*, *ureB*, *ureA*.
Allantoin, purine and glycosylate metabolism	16	2.01%	*all*, *allA*, *allB*, *allC*, *allD*, *allR*, *allS*, *ybbW* *ybbY*, *ylbE*, *glxK*, *glxR*, *fdrA*, *hyi*, *ylbF*, *arcC*.

Abbreviations: LPS, lipopolysaccharide; hvKp, hypervirulent. *Klebsiella pneumoniae* The number (No.) reflects the number of included studies in which at least one gene from each functional group was reported. Percentages (%) indicate the relative frequency of reporting of each functional group across the included studies. Gene names include canonical genes as well as reported variants or homologs, as described in the original studies.

**Table 6:** Comparative analysis of selected virulence-associated genes between classical (cKp) and hypervirulent (hvKp) *Klebsiella pneumoniae* strains.

Gene	cKp n/N (%)	*hvKp* n/N (%)	Absolute Difference (%)	95% CI	*p* Value (*Fisher*)
*rmpA*	238/450 (52.89)	346/436 (79.36)	26.47	(20.50–32.73)	<0.0001
*rmpA2*	162/450 (36.00)	256/436 (58.72)	22.72	(16.32–29.39)	<0.0001
*mrkD*	166/450 (36.89)	144/436 (33.03)	3.86	(−2.58–10.25)	0.2320
*iucA*	150/450 (33.33)	247/436 (56.65)	23.32	(16.96–29.97)	<0.0001
*iutA*	154/450 (34.22)	205/436 (47.02)	12.8	(6.32–19.42)	0.0001

Italic text indicates gene symbols.

**Table 7:** Comparative detection of selected virulence-associated genes according to molecular method in *K. pneumoniae* studies.

Gene	PCR-Based Methods n/N (%)	Sequencing-Based Methods n/N (%)	Absolute Difference (%)	95% CI	*p* Value (*Fisher*)
*rmpA*	205/305 (67.21)	215/371 (57.95)	9.26	(1.71 to 16.61)	0.0137
*rmpA2*	105/305 (34.43)	190/371 (51.21)	−16.79	(−24.48 to −9.40)	<0.0001
*mrkD*	133/305 (43.61)	115/371 (31.00)	12.61	(5.08 to 20.00)	0.0008
*iucA*	106/305 (34.75)	183/371 (49.33)	−14.58	(−22.26 to −7.16)	0.0002
*iutA*	92/305 (30.16)	186/371 (50.13)	−19.97	(−27.57 to −12.74)	<0.0001

Italic text indicates gene symbols.

## Data Availability

The data presented in this study are available in the article.

## Supplementary Materials

The following supporting information can be downloaded at https://www.mdpi.com/article/10.3390/pathogens15050556/s1: Table S1. PRISMA checklist; Table S2. Database search equations; Table S3. Data matrix for statistical analysis; Table S4. Justification of methodological quality; Table S5. Results of methodological quality assessment; Table S6. Data extracted from the studies; Table S7. Information on excluded and included subjects.
