# Supplementary material for: Genetic Fingerprint of Klebsiella pneumoniae Virulence: A Systematic Review
Source: Pathogens. 2026 May 21;15(5):556. doi: 10.3390/pathogens15050556 (PMC13209262; doi:10.3390/pathogens15050556)
Supplement: Supplementary file 1 [file pathogens-15-00556-s001.zip › Database_search_tableS2.pdf]

Database search

Google Academic:

("*Klebsiella pneumoniae*" OR "*K. pneumoniae*") AND ("Virulence" OR "Virulence genes" OR "Virulence factors" OR "Pathogenicity" OR "Virulence determinants" OR "Hypermucoviscosity") AND ("Molecular techniques" OR "Molecular methods" OR "Molecular epidemiology" OR "Genome sequencing" OR "Genotyping" OR "Molecular characterization")

Approximately 17,800 results (0.13 s)

allintitle: ("*Klebsiella pneumoniae*" OR "*K. pneumoniae*") AND ("Virulence" OR "Virulence genes" OR "Virulence factors" OR "Pathogenicity" OR "Virulence determinants" OR "Hypermucoviscosity") AND ("Molecular techniques" OR "Molecular methods" OR "Molecular epidemiology" OR "Genome sequencing" OR "Genotyping" OR "Molecular characterization")

Approximately 40 results (0.04 s)

inabstract: ("*Klebsiella pneumoniae*" OR "*K. pneumoniae*") AND ("Virulence" OR "Virulence genes" OR "Virulence factors" OR "Pathogenicity" OR "Virulence determinants" OR "Hypermucoviscosity") AND ("Molecular techniques" OR "Molecular methods" OR "Molecular epidemiology" OR "Genome sequencing" OR "Genotyping" OR "Molecular characterization")

Approximately 59 results (0.24 s)

("*Klebsiella pneumoniae*" OR "*K. pneumoniae*") AND ("Capsule" OR "Capsular polysaccharide" OR "CPS" OR "K antigen") AND ("PCR" OR "Multiplex PCR" OR "qPCR" OR "Real-time PCR" OR "Microarrays" OR "Whole genome sequencing" OR "WGS" OR "Next-generation sequencing" OR "NGS" OR "PFGE" OR "Pulsed-field gel electrophoresis")

Approximately 16,800 results (0.15 s)

allintitle: ("*Klebsiella pneumoniae*" OR "*K. pneumoniae*") AND ("Capsule" OR "Capsular polysaccharide" OR "CPS" OR "K antigen") AND ("PCR" OR "Multiplex PCR" OR "qPCR" OR "Real-time PCR" OR "Microarrays" OR "Whole genome sequencing" OR "WGS" OR "Next-generation sequencing" OR "NGS" OR "PFGE" OR "Pulsed-field gel electrophoresis")

1 result (0.08 s)

inabstract: ("*Klebsiella pneumoniae*" OR "*K. pneumoniae*") AND ("Capsule" OR "Capsular polysaccharide" OR "CPS" OR "K antigen") AND ("PCR" OR "Multiplex PCR" OR "qPCR"

OR "Real-time PCR" OR “Microarrays” OR "Whole genome sequencing" OR "WGS" OR "Next-generation sequencing" OR "NGS" OR "PFGE" OR "Pulsed-field gel electrophoresis")

Approximately 20 results (0.19 s)

("*Klebsiella pneumoniae*" OR "*K. pneumoniae*") AND ("Fimbriae" OR "Type 1 fimbriae" OR "Type 3 fimbriae" OR "Adhesins") AND ("PCR" OR "Multiplex PCR" OR “qPCR" OR "Real-time PCR" OR “Microarrays” OR "Whole genome sequencing" OR "WGS" OR "Next-generation sequencing" OR "NGS" OR "PFGE" OR "Pulsed-field gel electrophoresis")

Approximately 12,100 results (0.11 s)

allintitle: ("*Klebsiella pneumoniae*" OR "*K. pneumoniae*") AND ("Fimbriae" OR "Type 1 fimbriae" OR "Type 3 fimbriae" OR "Adhesins") AND ("PCR" OR "Multiplex PCR" OR “qPCR" OR "Real-time PCR" OR “Microarrays” OR "Whole genome sequencing" OR "WGS" OR "Next-generation sequencing" OR "NGS" OR "PFGE" OR "Pulsed-field gel electrophoresis")

0 results

inabstract: ("*Klebsiella pneumoniae*" OR "*K. pneumoniae*") AND ("Fimbriae" OR "Type 1 fimbriae" OR "Type 3 fimbriae" OR "Adhesins") AND ("PCR" OR "Multiplex PCR" OR "qPCR" OR "Real-time PCR" OR “Microarrays” OR "Whole genome sequencing" OR "WGS" OR "Next-generation sequencing" OR "NGS" OR "PFGE" OR "Pulsed-field gel electrophoresis")

Approximately 15 results (0.12 s)

("*Klebsiella pneumoniae*" OR "*K. pneumoniae*") AND ("Siderophores" OR "Enterobactin" OR "Aerobactin" OR "Yersiniabactin" OR "Salmochelin") AND ("PCR" OR "Multiplex PCR" OR "qPCR" OR "Real-time PCR" OR “Microarrays” OR "Whole genome sequencing" OR "WGS" OR "Next-generation sequencing" OR "NGS" OR "PFGE" OR "Pulsed-field gel electrophoresis")

Approximately 10,000 results (0.09 s)

allintitle: ("*Klebsiella pneumoniae*" OR "*K. pneumoniae*") AND ("Siderophores" OR "Enterobactin" OR "Aerobactin" OR "Yersiniabactin" OR "Salmochelin") AND ("PCR" OR "Multiplex PCR" OR “qPCR" OR "Real-time PCR" OR “Microarrays” OR "Whole genome sequencing" OR "WGS" OR "Next-generation sequencing" OR "NGS" OR "PFGE" OR "Pulsed-field gel electrophoresis")

0 results

inabstract: ("*Klebsiella pneumoniae*" OR "*K. pneumoniae*") AND ("Siderophores" OR "Enterobactin" OR "Aerobactin" OR "Yersiniabactin" OR "Salmochelin") AND ("PCR" OR "Multiplex PCR" OR "qPCR" OR "Real-time PCR" OR "Microarrays" OR "Whole genome sequencing" OR "WGS" OR "Next-generation sequencing" OR "NGS" OR "PFGE" OR "Pulsed-field gel electrophoresis")

Approximately 10 results (0.14 s)

("*Klebsiella pneumoniae*" OR "*K. pneumoniae*") AND ("rmpA" OR " OR "magA") "AND ("PCR" OR "Multiplex PCR" OR "qPCR" OR "Real-time PCR" OR "Microarrays" OR "Whole genome sequencing" OR "WGS" OR "Next generation sequencing" OR "NGS" OR "PFGE" OR "Pulsed field gel electrophoresis")

Approximately 3,190 results (0.13 s)

allintitle: ("*Klebsiella pneumoniae*" OR "*K. pneumoniae*") AND ("rmpA" OR "rmpA2" OR "magA) "AND ("PCR" OR "Multiplex PCR" OR "qPCR" OR "Real-time PCR" OR "Microarrays" OR "Whole genome sequencing" OR "WGS" OR "Next-generation sequencing" OR "NGS" OR "PFGE" OR "Pulsed-field gel electrophoresis")

3 results (0.07 s)

inabstract: ("*Klebsiella pneumoniae*" OR "*K. pneumoniae*") AND ("rmpA" OR "rmpA2" OR "magA) "AND ("PCR" OR "Multiplex PCR" OR "qPCR" OR "Real-time PCR" OR "Microarrays" OR "Whole genome sequencing" OR "WGS" OR "Next-generation sequencing" OR "NGS" OR "PFGE" OR "Pulsed-field gel electrophoresis")

2 results (0.07 s)

("*Klebsiella pneumoniae*" OR "*K. pneumoniae*") AND ("Biofilm formation" OR "Biofilm") AND ("PCR" OR "Multiplex PCR" OR "qPCR" OR "Real-time PCR" OR "Microarrays" OR "Whole genome sequencing" OR "WGS" OR "Next-generation sequencing" OR "NGS" OR "PFGE" OR "Pulsed-field gel electrophoresis")

Approximately 18,000 results (**0.07 s**)

allintitle: ("*Klebsiella pneumoniae*" OR "*K. pneumoniae*") AND ("Biofilm formation" OR "Biofilm") AND ("PCR" OR "Multiplex PCR" OR "qPCR" OR "Real-time PCR" OR "Microarrays" OR "Whole genome sequencing" OR "WGS" OR "Next-generation sequencing" OR "NGS" OR "PFGE" OR "Pulsed-field gel electrophoresis")

0 results

inabstract: ("*Klebsiella pneumoniae*" OR "*K. pneumoniae*") AND ("Biofilm formation" OR "Biofilm") AND ("PCR" OR "Multiplex PCR" OR “qPCR" OR "Real-time PCR" OR “Microarrays” OR "Whole genome sequencing" OR "WGS" OR "Next-generation sequencing" OR "NGS" OR "PFGE" OR "Pulsed-field gel electrophoresis")

Approximately 44 results (0.18 s)

("*Klebsiella pneumoniae*" OR "*K. pneumoniae*") AND ("Lipopolysaccharide" OR "LPS" OR "O antigen") AND ("PCR" OR "Multiplex PCR" OR “qPCR" OR "Real-time PCR" OR “Microarrays” OR "Whole genome sequencing" OR "WGS" OR "Next-generation sequencing" OR "NGS" OR "PFGE" OR "Pulsed-field gel electrophoresis")

Approximately 17,400 results (0.17 s)

allintitle: ("*Klebsiella pneumoniae*" OR "*K. pneumoniae*") AND ("Lipopolysaccharide" OR "LPS" OR "O antigen") AND ("PCR" OR "Multiplex PCR" OR “qPCR" OR "Real-time PCR" OR “Microarrays” OR "Whole genome sequencing" OR "WGS" OR "Next-generation sequencing" OR "NGS" OR "PFGE" OR "Pulsed-field gel electrophoresis")

3 results (0.07 s)

inabstract: ("*Klebsiella pneumoniae*" OR "*K. pneumoniae*") AND ("Lipopolysaccharide" OR "LPS" OR "O antigen") AND ("PCR" OR "Multiplex PCR" OR “qPCR" OR "Real-time PCR" OR “Microarrays” OR "Whole genome sequencing" OR "WGS" OR "Next-generation sequencing" OR "NGS" OR "PFGE" OR "Pulsed-field gel electrophoresis")

Approximately 31 results (0.19 s)

("*Klebsiella pneumoniae*" OR "*K. pneumoniae*") AND ("Efflux pumps") AND ("PCR" OR "Multiplex PCR" OR “qPCR" OR "Real-time PCR" OR “Microarrays” OR "Whole genome sequencing" OR "WGS" OR "Next-generation sequencing" OR "NGS" OR "PFGE" OR "Pulsed-field gel electrophoresis")

Approximately 15,400 results (0.11 s)

allintitle: ("*Klebsiella pneumoniae*" OR "*K. pneumoniae*") AND ("Efflux pumps") AND ("PCR" OR "Multiplex PCR" OR “qPCR" OR "Real-time PCR" OR “Microarrays” OR "Whole genome sequencing" OR "WGS" OR "Next-generation sequencing" OR "NGS" OR "PFGE" OR "Pulsed-field gel electrophoresis")

0 results (0.07 s)

inabstract: ("*Klebsiella pneumoniae*" OR "*K. pneumoniae*") AND ("Efflux pumps") AND ("PCR" OR "Multiplex PCR" OR “qPCR" OR "Real-time PCR" OR “Microarrays” OR "Whole genome

sequencing" OR "WGS" OR "Next-generation sequencing" OR "NGS" OR "PFGE" OR "Pulsed-field gel electrophoresis")

Approximately 24 results (0.10 s)

PUBMED

(("*Klebsiella pneumoniae*"[All Fields] OR "*K. pneumoniae*"[All Fields])) AND (("Virulence"[All Fields] OR "Virulence genes"[All Fields] OR "Virulence factors"[All Fields] OR "Pathogenicity"[All Fields] OR "Virulence determinants"[All Fields] OR "Hypermucoviscosity"[All Fields])) AND (("Molecular techniques"[All Fields] OR "Molecular methods"[All Fields] OR "Molecular epidemiology"[All Fields] OR "Genome sequencing"[All Fields] OR "Genotyping"[All Fields] OR "Molecular characterization"[All Fields]))

908 results

((("*Klebsiella pneumoniae*"[Title/Abstract] OR "*K. pneumoniae*"[Title/Abstract])) AND (("Virulence"[Title/Abstract] OR "Virulence genes"[Title/Abstract] OR "Virulence factors"[Title/Abstract] OR "Pathogenicity"[Title/Abstract] OR "Virulence determinants"[Title/Abstract] OR "Hypermucoviscosity"[Title/Abstract]))) AND (("Molecular techniques"[Title/Abstract] OR "Molecular methods"[Title/Abstract] OR "Molecular epidemiology"[Title/Abstract] OR "Genome sequencing"[Title/Abstract] OR "Genotyping"[Title/Abstract] OR "Molecular characterization"[Title/Abstract]))

772 results

(("*Klebsiella pneumoniae*"[All Fields] OR "*K. pneumoniae*"[All Fields])) AND (("Capsule"[All Fields] OR "Capsular polysaccharide"[All Fields] OR "CPS"[All Fields] OR "K antigen"[All Fields])) AND (("Molecular techniques"[All Fields] OR "Molecular methods"[All Fields] OR "Molecular epidemiology"[All Fields] OR "Genome sequencing"[All Fields] OR "Genotyping"[All Fields] OR "Molecular characterization"[All Fields]))

186 results

((("*Klebsiella pneumoniae*"[Title/Abstract] OR "*K. pneumoniae*"[Title/Abstract])) AND (("Capsule"[Title/Abstract] OR "Capsular polysaccharide"[Title/Abstract] OR "CPS"[Title/Abstract] OR "K antigen"[Title/Abstract]))) AND (("Molecular techniques"[Title/Abstract] OR "Molecular methods"[Title/Abstract] OR "Molecular epidemiology"[Title/Abstract] OR "Genome sequencing"[Title/Abstract] OR "Genotyping"[Title/Abstract] OR "Molecular characterization"[Title/Abstract]))

164 results

(("*Klebsiella pneumoniae*"[All Fields] OR "*K. pneumoniae*"[All Fields])) AND (("Fimbriae"[All Fields] OR "Type 1 fimbriae"[All Fields] OR "Type 3 fimbriae"[All Fields] OR "Adhesins"[All Fields])) AND (("PCR"[All Fields] OR "Multiplex PCR"[All Fields] OR "qPCR"[All Fields] OR "Real-time PCR"[All Fields] OR "Microarrays"[All Fields] OR "Whole genome sequencing"[All Fields] OR "WGS"[All Fields] OR "Next-generation sequencing"[All Fields] OR "NGS"[All Fields] OR "PFGE"[All Fields] OR "Pulsed-field gel electrophoresis"[All Fields]))

93 results

((("*Klebsiella pneumoniae*"[Title/Abstract] OR "*K. pneumoniae*"[Title/Abstract])) AND (("Fimbriae"[Title/Abstract] OR "Type 1 fimbriae"[Title/Abstract] OR "Type 3 fimbriae"[Title/Abstract] OR "Adhesins"[Title/Abstract]))) AND (("PCR"[Title/Abstract] OR "Multiplex PCR"[Title/Abstract] OR "qPCR"[Title/Abstract] OR "Real-time PCR"[Title/Abstract] OR "Microarrays"[Title/Abstract] OR "Whole genome sequencing"[Title/Abstract] OR "WGS"[Title/Abstract] OR "Next-generation sequencing"[Title/Abstract] OR "NGS"[Title/Abstract] OR "PFGE"[Title/Abstract] OR "Pulsed-field gel electrophoresis"[Title/Abstract]))

87 results

(("*Klebsiella pneumoniae*"[All Fields] OR "*K. pneumoniae*"[All Fields])) AND (("Siderophores"[All Fields] OR "Enterobactin"[All Fields] OR "Aerobactin"[All Fields] OR "Yersiniabactin"[All Fields] OR "Salmochelin"[All Fields])) AND (("PCR"[All Fields] OR "Multiplex PCR"[All Fields] OR "qPCR"[All Fields] OR "Real-time PCR"[All Fields] OR "Microarrays"[All Fields] OR "Whole genome sequencing"[All Fields] OR "WGS"[All Fields] OR "Next-generation sequencing"[All Fields] OR "NGS"[All Fields] OR "PFGE"[All Fields] OR "Pulsed-field gel electrophoresis"[All Fields]))

245 results

((("*Klebsiella pneumoniae*"[Title/Abstract] OR "*K. pneumoniae*"[Title/Abstract])) AND (("Siderophores"[Title/Abstract] OR "Enterobactin"[Title/Abstract] OR "Aerobactin"[Title/Abstract] OR "Yersiniabactin"[Title/Abstract] OR "Salmochelin"[Title/Abstract]))) AND (("PCR"[Title/Abstract] OR "Multiplex PCR"[Title/Abstract] OR "qPCR"[Title/Abstract] OR "Real-time PCR"[Title/Abstract] OR "Microarrays"[Title/Abstract] OR "Whole genome sequencing"[Title/Abstract] OR "WGS"[Title/Abstract] OR "Next-generation sequencing"[Title/Abstract] OR "NGS"[Title/Abstract] OR "PFGE"[Title/Abstract] OR "Pulsed-field gel electrophoresis"[Title/Abstract]))

223 results

(("*Klebsiella pneumoniae*"[All Fields] OR "*K. pneumoniae*"[All Fields]) AND (("*rmp*A"[All Fields] OR "*rmp*A2"[All Fields] OR "*mag*A"[All Fields])) AND (("PCR"[All Fields] OR "Multiplex PCR"[All Fields] OR "qPCR"[All Fields] OR "Real-time PCR"[All Fields] OR "Microarrays"[All Fields] OR "Whole genome sequencing"[All Fields] OR "WGS"[All Fields] OR "Next-generation sequencing"[All Fields] OR "NGS"[All Fields] OR "PFGE"[All Fields] OR "Pulsed-field gel electrophoresis"[All Fields]))

320 results

((("*Klebsiella pneumoniae*"[Title/Abstract] OR "*K. pneumoniae*"[Title/Abstract])) AND (("*rmp*A"[Title/Abstract] OR "*rmp*A2"[Title/Abstract] OR "*mag*A"[Title/Abstract]))) AND (("PCR"[Title/Abstract] OR "Multiplex PCR"[Title/Abstract] OR "qPCR"[Title/Abstract] OR "Real-time PCR"[Title/Abstract] OR "Microarrays"[Title/Abstract] OR "Whole genome sequencing"[Title/Abstract] OR "WGS"[Title/Abstract] OR "Next-generation sequencing"[Title/Abstract] OR "NGS"[Title/Abstract] OR "PFGE"[Title/Abstract] OR "Pulsed-field gel electrophoresis"[Title/Abstract]))

314 results

(("*Klebsiella pneumoniae*"[All Fields] OR "*K. pneumoniae*"[All Fields])) AND (("Biofilm formation"[All Fields] OR "Biofilm"[All Fields])) AND (("PCR"[All Fields] OR "Multiplex PCR"[All Fields] OR "qPCR"[All Fields] OR "Real-time PCR"[All Fields] OR "Microarrays"[All Fields] OR "Whole genome sequencing"[All Fields] OR "WGS"[All Fields] OR "Next-generation sequencing"[All Fields] OR "NGS"[All Fields] OR "PFGE"[All Fields] OR "Pulsed-field gel electrophoresis"[All Fields]))

312 results

((("*Klebsiella pneumoniae*"[Title/Abstract] OR "*K. pneumoniae*"[Title/Abstract])) AND (("Biofilm formation"[Title/Abstract] OR "Biofilm"[Title/Abstract]))) AND (("PCR"[Title/Abstract] OR "Multiplex PCR"[Title/Abstract] OR "qPCR"[Title/Abstract] OR "Real-time PCR"[Title/Abstract] OR "Microarrays"[Title/Abstract] OR "Whole genome sequencing"[Title/Abstract] OR "WGS"[Title/Abstract] OR "Next-generation sequencing"[Title/Abstract] OR "NGS"[Title/Abstract] OR "PFGE"[Title/Abstract] OR "Pulsed-field gel electrophoresis"[Title/Abstract]))

301 results

(("*Klebsiella pneumoniae*"[All Fields] OR "*K. pneumoniae*"[All Fields])) AND (("Lipopolysaccharide"[All Fields] OR "LPS"[All Fields] OR "O antigen"[All Fields])) AND

(("PCR"[All Fields] OR "Multiplex PCR"[All Fields] OR "qPCR"[All Fields] OR "Real-time PCR"[All Fields] OR "Microarrays"[All Fields] OR "Whole genome sequencing"[All Fields] OR "WGS"[All Fields] OR "Next-generation sequencing"[All Fields] OR "NGS"[All Fields] OR "PFGE"[All Fields] OR "Pulsed-field gel electrophoresis"[All Fields]))

87 results

((("*Klebsiella pneumoniae*"[Title/Abstract] OR "*K. pneumoniae*"[Title/Abstract])) AND (("Lipopolysaccharide"[Title/Abstract] OR "LPS"[Title/Abstract] OR "O antigen"[Title/Abstract]))) AND (("PCR"[Title/Abstract] OR "Multiplex PCR"[Title/Abstract] OR "qPCR"[Title/Abstract] OR "Real-time PCR"[Title/Abstract] OR "Microarrays"[Title/Abstract] OR "Whole genome sequencing"[Title/Abstract] OR "WGS"[Title/Abstract] OR "Next-generation sequencing"[Title/Abstract] OR "NGS"[Title/Abstract] OR "PFGE"[Title/Abstract] OR "Pulsed-field gel electrophoresis"[Title/Abstract]))

80 results

(("*Klebsiella pneumoniae*"[All Fields] OR "*K. pneumoniae*"[All Fields])) AND (("Efflux pumps"[All Fields])) AND (("PCR"[All Fields] OR "Multiplex PCR"[All Fields] OR "qPCR"[All Fields] OR "Real-time PCR"[All Fields] OR "Microarrays"[All Fields] OR "Whole genome sequencing"[All Fields] OR "WGS"[All Fields] OR "Next-generation sequencing"[All Fields] OR "NGS"[All Fields] OR "PFGE"[All Fields] OR "Pulsed-field gel electrophoresis"[All Fields]))

96 results

((("*Klebsiella pneumoniae*"[Title/Abstract] OR "*K. pneumoniae*"[Title/Abstract])) AND (("Efflux pumps"[Title/Abstract]))) AND (("PCR"[Title/Abstract] OR "Multiplex PCR"[Title/Abstract] OR "qPCR"[Title/Abstract] OR "Real-time PCR"[Title/Abstract] OR "Microarrays"[Title/Abstract] OR "Whole genome sequencing"[Title/Abstract] OR "WGS"[Title/Abstract] OR "Next-generation sequencing"[Title/Abstract] OR "NGS"[Title/Abstract] OR "PFGE"[Title/Abstract] OR "Pulsed-field gel electrophoresis"[Title/Abstract]))

96 results

ScienceDirect

("*Klebsiella pneumoniae*") AND ("Virulence" OR "Virulence genes") AND ("Molecular techniques" OR "Molecular methods" OR "Molecular epidemiology" OR "Genome sequencing" OR "Genotyping" OR "Molecular characterization")

244 results

Title, abstract, keywords: ("*Klebsiella pneumoniae*") AND ("Capsule" OR "Capsular polysaccharide") AND ("Multiplex PCR" OR "qPCR" OR "Real-time PCR" OR "Whole genome sequencing" OR "Next-generation sequencing" OR "Pulsed-field gel electrophoresis")

44 results

Title, abstract, keywords: ("*Klebsiella pneumoniae*") AND ("Fimbriae" OR "Adhesins") AND ("Multiplex PCR" OR "qPCR" OR "Real-time PCR" OR "Whole genome sequencing" OR "Next-generation sequencing" OR "Pulsed-field gel electrophoresis")

14 results

Title, abstract, keywords: ("*Klebsiella pneumoniae*") AND ("Type 1 fimbriae" OR " Type 3 fimbriae") AND ("Multiplex PCR" OR "qPCR" OR "Real-time PCR" OR "Whole genome sequencing" OR "Next-generation sequencing" OR "Pulsed-field gel electrophoresis")

13 results

Title, abstract, keywords: ("*Klebsiella pneumoniae*") AND ("Yersiniabactin" OR "Salmochelin") AND ("Multiplex PCR" OR "qPCR" OR "Real-time PCR" OR "Whole genome sequencing" OR "Next-generation sequencing" OR "Pulsed-field gel electrophoresis")

23 results

Title, abstract, keywords: ("*Klebsiella pneumoniae*") AND ("Siderophores" OR "Enterobactin") AND ("Multiplex PCR" OR "qPCR" OR "Real-time PCR" OR "Whole genome sequencing" OR "Next-generation sequencing" OR "Pulsed-field gel electrophoresis")

34 results

Title, abstract, keywords: ("*Klebsiella pneumoniae*") AND (“Aerobactin") AND ("Multiplex PCR" OR "qPCR" OR "Real-time PCR" OR "Whole genome sequencing" OR "Next-generation sequencing" OR "Pulsed-field gel electrophoresis")

20 results

Title, abstract, keywords: ("*Klebsiella pneumoniae*") AND (“*rmp*A" OR "*mag*A") AND ("Multiplex PCR" OR "qPCR" OR "Real-time PCR" OR "Whole genome sequencing" OR "Next-generation sequencing" OR "Pulsed-field gel electrophoresis")

41 results

Title, abstract, keywords: ("*Klebsiella pneumoniae*") AND (“*rmp*A2" OR "*mag*A") AND ("Multiplex PCR" OR "qPCR" OR "Real-time PCR" OR "Whole genome sequencing" OR "Next-generation sequencing" OR "Pulsed-field gel electrophoresis")

29 results

("*Klebsiella pneumoniae*" OR "*K. pneumoniae*") AND ("Biofilm formation" OR "Biofilm") AND ("Multiplex PCR" OR "qPCR" OR "Real-time PCR" OR "Whole genome sequencing" OR "Next-generation sequencing" OR "Pulsed-field gel electrophoresis")

2,247 results

Title, abstract, keywords: ("*Klebsiella pneumoniae*" OR "*K. pneumoniae*") AND ("Biofilm formation" OR "Biofilm") AND ("Multiplex PCR" OR "qPCR" OR "Real-time PCR" OR "Whole genome sequencing" OR "Next-generation sequencing" OR "Pulsed-field gel electrophoresis")

43 results

("*Klebsiella pneumoniae*" OR "*K. pneumoniae*") AND ("LPS" OR " Lipopolysaccharide ") AND ("Multiplex PCR" OR "qPCR" OR "Real-time PCR" OR "Whole genome sequencing" OR "Next-generation sequencing" OR "Pulsed-field gel electrophoresis")

1.977 results

Title, abstract, keywords: ("*Klebsiella pneumoniae*" OR "*K. pneumoniae*") AND ("LPS" OR " Lipopolysaccharide ") AND ("Multiplex PCR" OR "qPCR" OR "Real-time PCR" OR "Whole genome sequencing" OR "Next-generation sequencing" OR "Pulsed-field gel electrophoresis")

5 results

Title, abstract, keywords: ("*Klebsiella pneumoniae*" OR "*K. pneumoniae*") AND ("LPS" OR "O antigen") AND ("Multiplex PCR" OR "qPCR" OR "Real-time PCR" OR "Whole genome sequencing" OR "Next-generation sequencing" OR "Pulsed-field gel electrophoresis")

2 results

("*Klebsiella pneumoniae*" OR "*K. pneumoniae*") AND ("Efflux pumps") AND ("Multiplex PCR" OR "qPCR" OR "Real-time PCR" OR "Whole genome sequencing" OR "Next-generation sequencing" OR "Pulsed-field gel electrophoresis")

1,907 results

Title, abstract, keywords: ("*Klebsiella pneumoniae*" OR "*K. pneumoniae*") AND ("Efflux pumps") AND ("Multiplex PCR" OR "qPCR" OR "Real-time PCR" OR "Whole genome sequencing" OR "Next-generation sequencing" OR "Pulsed-field gel electrophoresis")

38 results

SCOPUS

ALL (((“*Klebsiella pneumoniae*" OR "*K. pneumoniae*”) AND (“Virulence" OR "Virulence genes" OR "Virulence factors" OR "Pathogenicity" OR "Virulence determinants" OR "Hypermucoviscosity”) AND (“Molecular techniques" OR "Molecular methods" OR "Molecular epidemiology" OR "Genome sequencing" OR "Genotyping" OR "Molecular characterization”)))

20,486 Documents

TITLE-ABS-KEY ("*Klebsiella pneumoniae*" OR "*K. pneumoniae*”) AND (“Virulence" OR "Virulence genes" OR "Virulence factors" OR "Pathogenicity" OR "Virulence determinants" OR "Hypermucoviscosity”) AND (“Molecular techniques" OR "Molecular methods" OR "Molecular epidemiology" OR "Genome sequencing" OR "Genotyping" OR "Molecular characterization"))

1640 Documents

ALL (("*Klebsiella pneumoniae*" OR "*K. pneumoniae*") AND ("Capsule" OR "Capsular polysaccharide" OR "CPS" OR "K antigen") AND ("PCR" OR "Multiplex PCR" OR "qPCR" OR "Real-time PCR" OR "Microarrays" OR "Whole genome sequencing" OR "WGS" OR "Next-generation sequencing" OR "NGS" OR "PFGE" OR "Pulsed-field gel electrophoresis")))

4,088 Documents

TITLE-ABS-KEY (("*Klebsiella pneumoniae*" OR "*K. pneumoniae*") AND ("Capsule" OR "Capsular polysaccharide" OR "CPS" OR "K antigen") AND ("PCR" OR "Multiplex PCR" OR "qPCR" OR "Real-time PCR" OR "Microarrays" OR "Whole genome sequencing" OR "WGS" OR "Next-generation sequencing" OR "NGS" OR "PFGE" OR "Pulsed-field gel electrophoresis"))

434 Documents

ALL ("*Klebsiella pneumoniae*" OR "*K. pneumoniae*") AND ("Fimbriae" OR "Type 1 fimbriae" OR "Type 3 fimbriae" OR "Adhesins") AND ("PCR" OR "Multiplex PCR" OR "qPCR" OR "Real-

time PCR" OR "Microarrays" OR "Whole genome sequencing" OR "WGS" OR "Next-generation sequencing" OR "NGS" OR "PFGE" OR "Pulsed-field gel electrophoresis")))

3,091 Documents

TITLE-ABS-KEY (("*Klebsiella pneumoniae*" OR "*K. pneumoniae*") AND ("Fimbriae" OR "Type 1 fimbriae" OR "Type 3 fimbriae" OR "Adhesins") AND ("PCR" OR "Multiplex PCR" OR "qPCR" OR "Real-time PCR" OR "Microarrays" OR "Whole genome sequencing" OR "WGS" OR "Next-generation sequencing" OR "NGS" OR "PFGE" OR "Pulsed-field gel electrophoresis"))

183 Documents

ALL ((("*Klebsiella pneumoniae*" OR "*K. pneumoniae*") AND (“Siderophores" OR "Enterobactin" OR "Aerobactin" OR "Yersiniabactin" OR "Salmochelin") AND ("PCR" OR "Multiplex PCR" OR "qPCR" OR "Real-time PCR" OR "Microarrays" OR "Whole genome sequencing" OR "WGS" OR "Next-generation sequencing" OR "NGS" OR "PFGE" OR "Pulsed-field gel electrophoresis")))

2,079 Documents

TITLE-ABS-KEY (("*Klebsiella pneumoniae*" OR "*K. pneumoniae*") AND ("Siderophores" OR "Enterobactin" OR "Aerobactin" OR "Yersiniabactin" OR "Salmochelin") AND ("PCR" OR "Multiplex PCR" OR "qPCR" OR "Real-time PCR" OR "Microarrays" OR "Whole genome sequencing" OR "WGS" OR "Next-generation sequencing" OR "NGS" OR "PFGE" OR "Pulsed-field gel electrophoresis"))

419 Documents

ALL (((“*Klebsiella pneumoniae*" OR "*K. pneumoniae*”) AND (“Biofilm formation" OR "Biofilm”) AND (“PCR" OR "Multiplex PCR" OR "qPCR" OR "Real-time PCR" OR "Microarrays" OR "Whole genome sequencing" OR "WGS" OR "Next-generation sequencing" OR "NGS" OR "PFGE" OR "Pulsed-field gel electrophoresis”)))

11,755 Documents

TITLE-ABS-KEY ((“*Klebsiella pneumoniae*" OR "*K. pneumoniae*”) AND (“Biofilm formation" OR "Biofilm”) AND (“PCR" OR "Multiplex PCR" OR "qPCR" OR "Real-time PCR" OR "Microarrays" OR "Whole genome sequencing" OR "WGS" OR "Next-generation sequencing" OR "NGS" OR "PFGE" OR "Pulsed-field gel electrophoresis”))

707 Documents

ALL ((“*Klebsiella pneumoniae*" OR "*K. pneumoniae*”) AND (“Lipopolysaccharide" OR "LPS" OR "O antigen”) AND (“PCR" OR "Multiplex PCR" OR "qPCR" OR "Real-time PCR" OR "Microarrays" OR "Whole genome sequencing" OR "WGS" OR "Next-generation sequencing" OR "NGS" OR "PFGE" OR "Pulsed-field gel electrophoresis”))

5,158 Documents

TITLE-ABS-KEY ((“*Klebsiella pneumoniae*" OR "*K. pneumoniae*”) AND (“Lipopolysaccharide" OR "LPS" OR "O antigen”) AND (“PCR" OR "Multiplex PCR" OR "qPCR" OR "Real-time PCR" OR "Microarrays" OR "Whole genome sequencing" OR "WGS" OR "Next-generation sequencing" OR "NGS" OR "PFGE" OR "Pulsed-field gel electrophoresis”))

222 Documents

ALL (((“*Klebsiella pneumoniae*" OR "*K. pneumoniae*”) AND (“*rmp*A" OR "*mag*A”) AND (“PCR" OR "Multiplex PCR" OR "qPCR" OR "Real-time PCR" OR "Microarrays" OR "Whole genome sequencing" OR "WGS" OR "Next generation sequencing" OR "NGS" OR "PFGE" OR "Pulsed field gel electrophoresis”)))

1,133 Documents

TITLE-ABS-KEY ((“*Klebsiella pneumoniae*" OR "*K. pneumoniae*”) AND (“*rmp*A" OR "*mag*A”) AND (“PCR" OR "Multiplex PCR" OR "qPCR" OR "Real-time PCR" OR "Microarrays" OR "Whole genome sequencing" OR "WGS" OR "Next generation sequencing" OR "NGS" OR "PFGE" OR "Pulsed field gel electrophoresis”))

414 Documents

ALL ((“*Klebsiella pneumoniae*" OR "*K. pneumoniae*”) AND (“Efflux pumps”) AND (“PCR" OR "Multiplex PCR" OR "qPCR" OR "Real-time PCR" OR "Microarrays" OR "Whole genome sequencing" OR "WGS" OR "Next-generation sequencing" OR "NGS" OR "PFGE" OR "Pulsed-field gel electrophoresis”))

5,105 Documents

TITLE-ABS-KEY ((“*Klebsiella pneumoniae*" OR "*K. pneumoniae*”) AND (“Efflux pumps”) AND (“PCR" OR "Multiplex PCR" OR "qPCR" OR "Real-time PCR" OR "Microarrays" OR "Whole genome sequencing" OR "WGS" OR "Next-generation sequencing" OR "NGS" OR "PFGE" OR "Pulsed-field gel electrophoresis”))

308 Documents
